# Supplementary material for: Discovery of New Uracil and Thiouracil Derivatives as Potential HDAC Inhibitors
Source: Pharmaceuticals (Basel). 2023 Jul 6;16(7):966. doi: 10.3390/ph16070966 (PMC10384246; doi:10.3390/ph16070966)
Supplement: Supplementary file 1 [file pharmaceuticals-16-00966-s001.zip › pharmaceuticals-2450172-supplementary.pdf]

Discovery of new uracil and thiouracil derivatives as potential HDAC inhibitors

Omnia R. Elbatrawy[1], Mohamed Hagras[2*], Moshira A. El Deeb[1], Fatimah Agili[3], Maghawry Hegazy[4], Ahmed A. El-Husseiny[4,5], Mahmoud Mohamed Mokhtar[4], Samy Y. Elkhawaga[4], Ibrahim H. Eissa[6*], Samar El-Kalyoubi[7*]

[1] Pharmaceutical Organic Chemistry Department, Faculty of Pharmacy (Girls), Al-Azhar University, Cairo, Egypt. Omniaelbatrawy2@gmail.com, Moshiraadeleldeeb.pharmg@azhar.edu.eg

[2] Pharmaceutical Organic Chemistry Department, Faculty of Pharmacy (Boys), Al-Azhar University, Cairo, Egypt. m.hagrs@azhar.edu.eg

[3] Chemistry Department, Faculty of Science (Female Section), Jazan University, Jazan 82621, Saudi Arabia. fatmah2000@gmail.com

[4] Biochemistry and Molecular Biology Department, Faculty of Pharmacy (Boys), Al-Azhar University, Cairo, Egypt. drmegawry@azhar.edu.eg, mahmoud.mokhtar@azhar.edu.eg, syussuf2012@azhar.edu.eg

[5] Department of Biochemistry, Faculty of Pharmacy, Egyptian Russian University, Badr City 11829, Cairo, Egypt. Ahmedabdullah1984@azhar.edu.eg

[6] Pharmaceutical Medicinal Chemistry & Drug Design Department, Faculty of Pharmacy (Boys), Al-Azhar University, Cairo, Egypt. ibrahimeissa@azhar.edu.eg

[7] Department of Pharmaceutical Organic Chemistry, Faculty of Pharmacy, Port Said University, 42511 Port Said, Egypt. s.elkalyoubi@hotmail.com

Corresponding authors

Samar El-Kalyoubi: Department of Pharmaceutical Organic Chemistry, Faculty of Pharmacy, Port Said University, 42511 Port Said, Egypt. s.elkalyoubi@hotmail.com

Mohamed Hagras: Pharmaceutical Organic Chemistry Department, Faculty of Pharmacy (Boys), Al-Azhar University, Cairo, Egypt. m.hagrs@azhar.edu.eg

Ibrahim H. Eissa: Pharmaceutical Medicinal Chemistry & Drug Design Department, Faculty of Pharmacy (Boys), Al-Azhar University, Cairo, Egypt. ibrahimeissa@azhar.edu.eg

Content

**1**	Chemistry
**2**	Biological tests
**3**	Docking studies
**4**	Spectral data
**5**	HPLC

S1. Chemistry

S1.1. General

Melting points were determined with a Gallenkamp (London, UK) melting point apparatus and were uncorrected. The proton nuclear magnetic resonance (1H NMR) spectra were recorded on Bruker 400 MHz Spectrometer and 13C NMR spectra were run at 125 MHz in dimethylsulfoxide (DMSO-d6) and tetramethylsilane (TMS) as an internal standard, Applied Nucleic Acid Research Center, Zagazig University, Egypt. The reaction progress was followed by thin-layer chromatography (TLC) using TLC sheets precoated with ultraviolet (UV) fluorescent silica gel (Merck 60F254) and spots were visualized by irradiation with UV light (254 nm). IR Spectrum, Mass Spec. and Microanalyses for C, H and N were performed at the Regional Center for Mycology and Biotechnology, Al-Azhar University. All the results of elemental analyses corresponded to the calculated values within experimental error. All starting materials and reagents were generally commercially available and purchased from Sigma-Aldrich or Alfa-Aesar.

S1.2. Chromatographic conditions

HPLC SYSTEM:

The analysis were performed by HPLC-(Agilent 1100) is composed of a two LC- pumps pump, a UV/Vis detector. C18 column (125 mm × 4.60 mm, 5 µm particle size).

Chromatograms were obtained and analyzed using the Agilent ChemStation

Mobile Phase :

Solvent A: Water with 1% formic acid; Solvent B: Acetonitrile with 1% formic acid.

Gradient Condition: 10% B and 90% A to 90% B and 10%A in 9 min.

90% B and 10% A to 10% B and 90%A in 9 min.

Flow Rate: 2 ml/ min

Detector: UV 254 nm

Injection solvent volume : 20 µl

S2. Biological tests

S2.1. In vitro anti-proliferative activities

The *in vitro* antiproliferative activities of all the synthesized compounds against three human tumor cell lines namely, MCF-7 (human breast cancer cell line), HepG2 (human liver carcinoma cell line), colorectal carcinoma (HCT-116) were evaluated quantitatively as described in the literature, using MTT assay protocol. Sorafenib was used in this test as a positive control. The anti-proliferative activity was assessed quantitatively as follows.

Human cancer cell lines were seeded in 96-well plates at a density of 3-8 × 10^3 cells/well. Next, the cells were incubated for 12 h in a 5% CO_2 incubator at 37 °C. Then, for each well, the growth medium was exchanged with 0.1 ml of fresh medium containing graded concentrations of the test compounds or equal DMSO. Then 10 µl MTT solution (5 µg/ml) was added to each well, and the cells were incubated for additional 4 h. The crystals of MTT-formazan were dissolved in 100 µl of DMSO; the absorbance of each well was measured at 490 nm using an automatic ELISA reader system (TECAN, CHE). The IC_{50} values were calculated using the nonlinear regression fitting models (Graph Pad, Prism Version 5). The data represented the mean of three independent experiments in triplicate and were expressed as means ± SD. The IC_{50} value was

defined as the concentration at which 50% of the cells could survive. The results were expressed as growth inhibitory concentration (IC_{50}) values and summarized in **Table 1**.

S2.2. In-vitro **DNA-Flow cytometric (cell cycle) analysis.**

HCT-116 cells were treated with the most active member **5m** at concentrations of 78 µM for 72 h. Then, the tested cells were collected by trypsinization and washed in PBS. Ice-cold absolute ethanol was used for fixation of the collected cells. The cells were stained with Cycle TESTTM PLUS DNA Reagent Kit (BD Biosciences, San Jose, CA) according to the manufacturer's instructions. Cell-cycle distribution was evaluated using a flow cytometer.

S2.3. Annexin V-FITC apoptosis assay.

To detect the apoptosis induced by compound **5m**, HCT-116 cells were seeded and incubated overnight and then treated with compound **5m** at concentrations of 78 µM for 72 h. DMSO was chosen as the negative control. After that, the cells were collected and washed with PBS two successive times with centrifugation. Apoptosis detection kit (BD Biosciences, San Jose, CA) was used in this test. According to the manufacturer's protocol the cells were stained by Annexin V-FITC and propidium iodide (PI) in the binding buffer for 20min at room temperature in the dark. Using a flow cytometer, Annexin V-FITC and PI binding were analyzed. Flowjo software was used to analyze the frequencies in all quadrants.

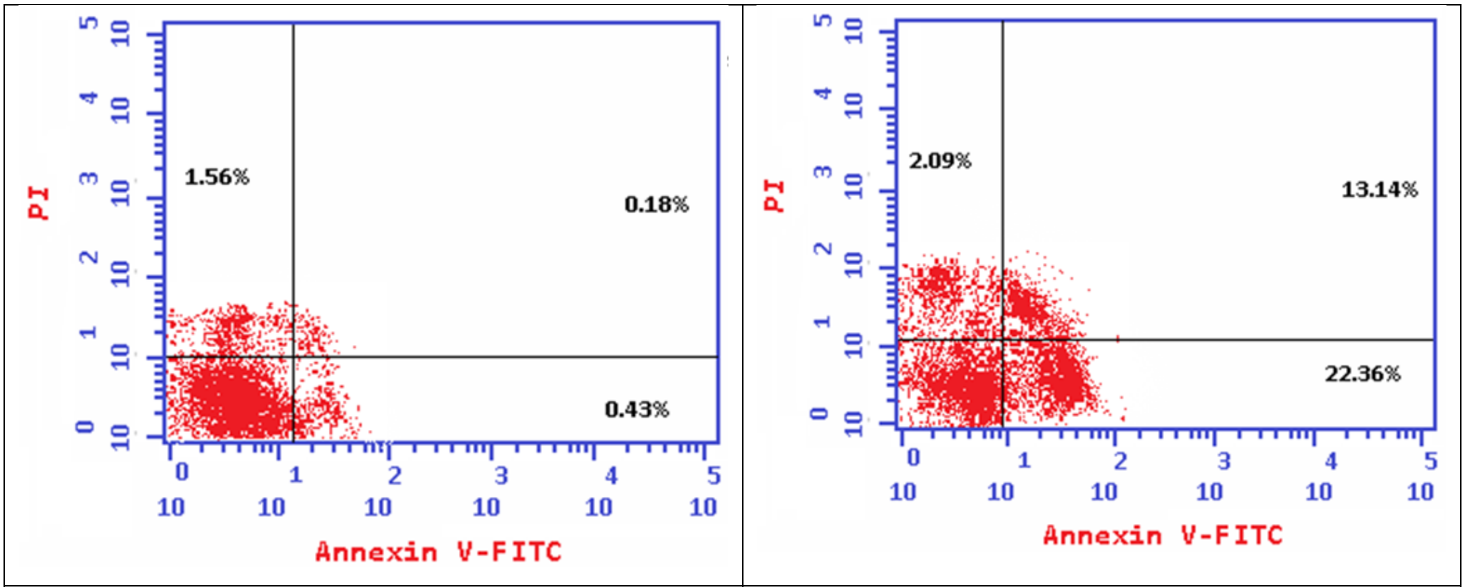

**A)** Control (HCT-116)	**B)** Compound **5m**

S2.4. Apoptotic effect of compound **5m** treatment. (**A**) Control (HCT-116) cells. (**B**) HCT-116 cells treated with compound **5m**.

S2.5. Quantitative Real Time Reverse-Transcriptase PCR technique (determination of caspase-3 and caspase-8)

1. ***Total RNA isolation:***

The total RNA was extracted from the control and treated cells by using RNeasy mini kit (Qiagen, Germany) according the manufacturer's protocol. Then, the isolated RNAs were quantitatively and qualitatively assessed using a NanoDrop®1000 spectrophotometer (Thermo Scientific, Wilmington, DE, USA). The RNA aliquots were preserved at - 80 °C till the next step[1].

2. **Reverse transcription and quantification of caspase-3 and caspase-8 mRNA expressions using qRT-PCR:**

iScript™ One-Step RT-PCR Kit with SYBR® Green kit (BIORAD Laboratories, USA) was used for reverse transcription of total RNA and quantification of the targeted mRNA expressions in single reaction tube. In depth, the complementary DNA (cDNA) was performed efficiently from the isolated RNA using iScript™ MMLV (Moloney Murine Leukemia Virus) reverse transcriptase. While, the relative expression levels of caspase-3 and caspase-8 mRNA were determined using 2X SYBR® Green RT-PCR Reaction Mix, containing iTaq™ DNA polymerase and SYBR® Green I dye, on the basis of the manufacturer's guidelines. GAPDH was used as housekeeping gene for data normalization. The reaction Mix consisted of 10 μL of 2X SYBR® Green RT-PCR Reaction Mix, 1 μl of forward primer, 1 μl of reverse primer, 1 μl of iScript Reverse Transcriptase for One-Step RT-PCR, 20 ng of the isolated total RNA and the reaction volume was completed to 20 μl with nuclease-free water according the manufacturer's instructions. The relative expression levels and fold change of caspase-3 and caspase-8 mRNAs were determined by using the $\mathbf{2^{-\Delta\Delta ct}}$ method.

S3. Docking studies

The crystal structure of the target enzyme HDAC1 (PDB ID: 1C3R, resolution 2.00 Å) was downloaded from Protein Data Bank (http://www.pdb.org). Molecular Operating Environment (MOE2019) was used for the docking analysis. In these studies, the free energies, and binding modes of the designed molecules against HDAC1 were determined. At first, the water molecules were removed from the crystal structure of HDAC1. Trichostatin A (The co-crystallized ligand) was utilized as a reference molecule in the docking process. After that, in order to prepare the target molecule for binding with the designed compounds, the target protein

was subjected to protonation step. Then, the hydrogen atoms were hidden to make the areas of interaction clearer. Next, the energy of the system was minimized followed by identification the binding pocket of the target protein.

The structures of the designed compounds and the co-crystallized ligand, trichostatin A were drawn using ChemBioDraw Ultra 14.0 and saved as SDF format. Then, the saved files were opened using MOE 2019 and 3D structures were protonated. Next, the energy of the molecules was minimized. Validation process was performed for each target by running the docking process for only the co-crystallized ligand. Low RMSD values between docked and crystal conformations indicate valid performance. The docking procedures were carried out utilizing a default protocol. In each case, 30 docked structures were generated using genetic algorithm searches. The output from of MOE2019 was further analyzed and visualized using Discovery Studio software.

S4. Spectral data

5,5'-(Phenylmethylene)bis(6-amino-1-ethyl-2-thioxo-2,3-dihydropyrimidin-4(1*H*)-one) (**5a)**

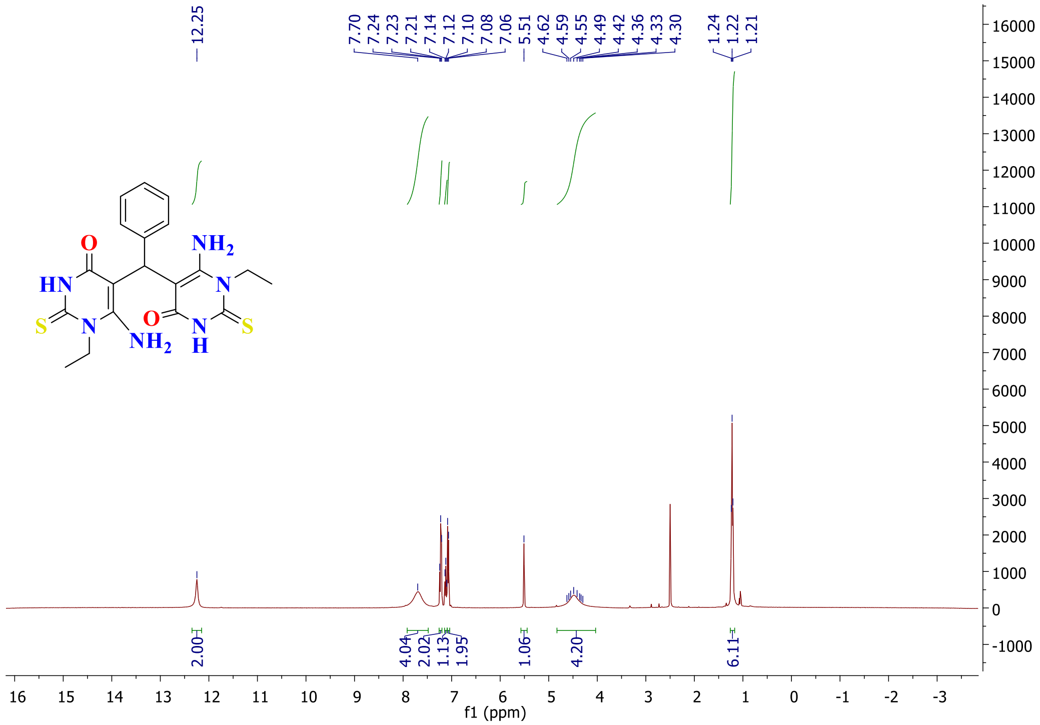

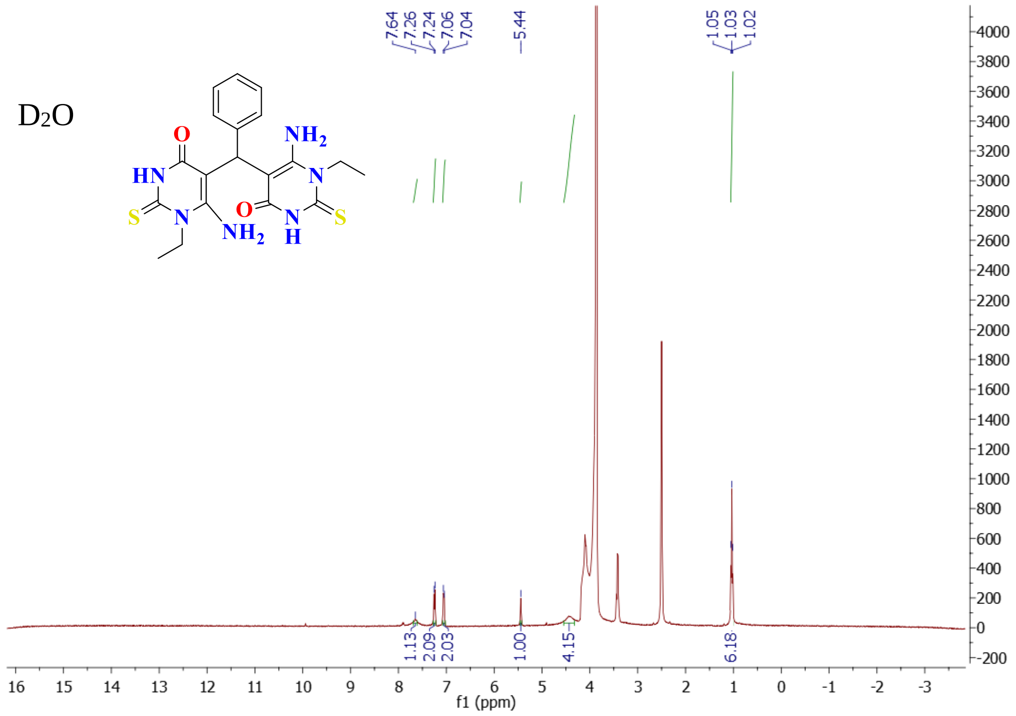

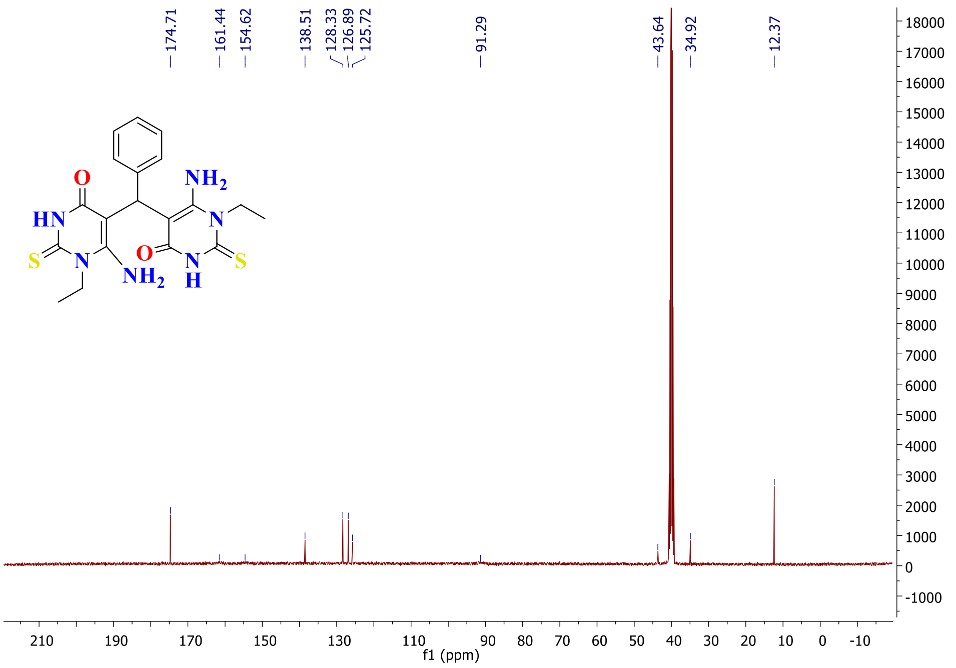

174.71
161.44
154.62
138.51
128.33
126.89
125.72
91.29
43.64
34.92
12.37
O
HN
S
N
NH2
NH2
N
O
N
H
S
18000
17000
16000
15000
14000
13000
12000
11000
10000
9000
8000
7000
6000
5000
4000
3000
2000
1000
0
-1000
210 190 170 150 130 110 90 80 70 60 50 40 30 20 10 0 -10
f1 (ppm)

5,5'-(*p*-tolylmethylene)bis(6-amino-1-ethyl-2-thioxo-2,3-dihydropyrimidin-4(1*H*)-one) (**5b)**

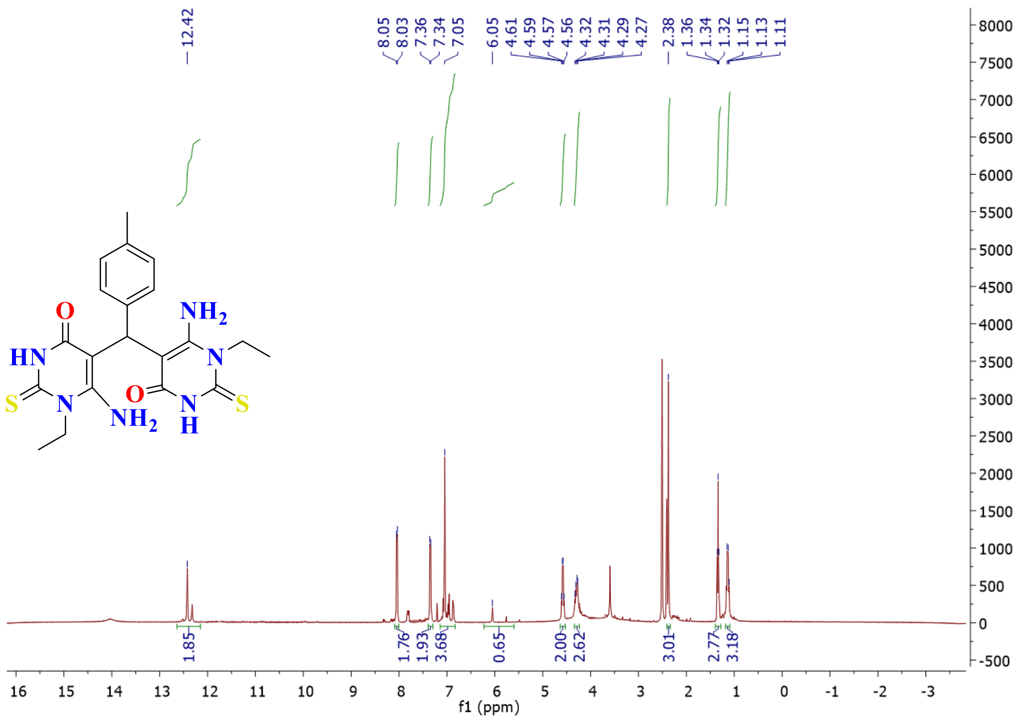

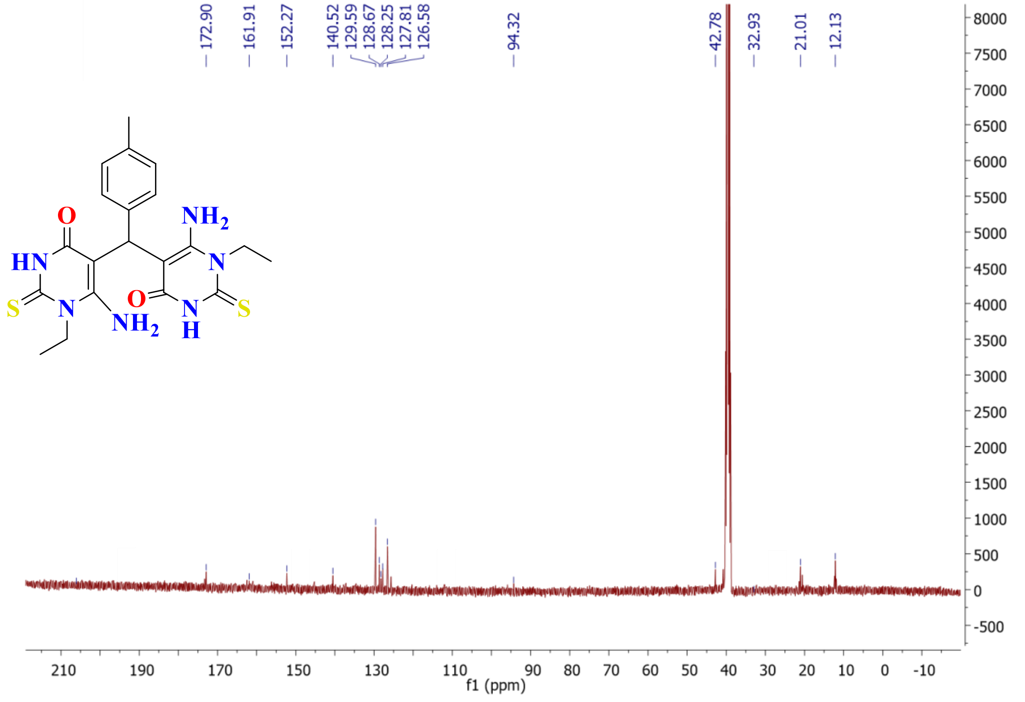

5,5'-((4-chlorophenyl)methylene)bis(6-amino-1-ethyl-2-thioxo-2,3-dihydropyrimidin-4(1*H*)-one) (**5c**)

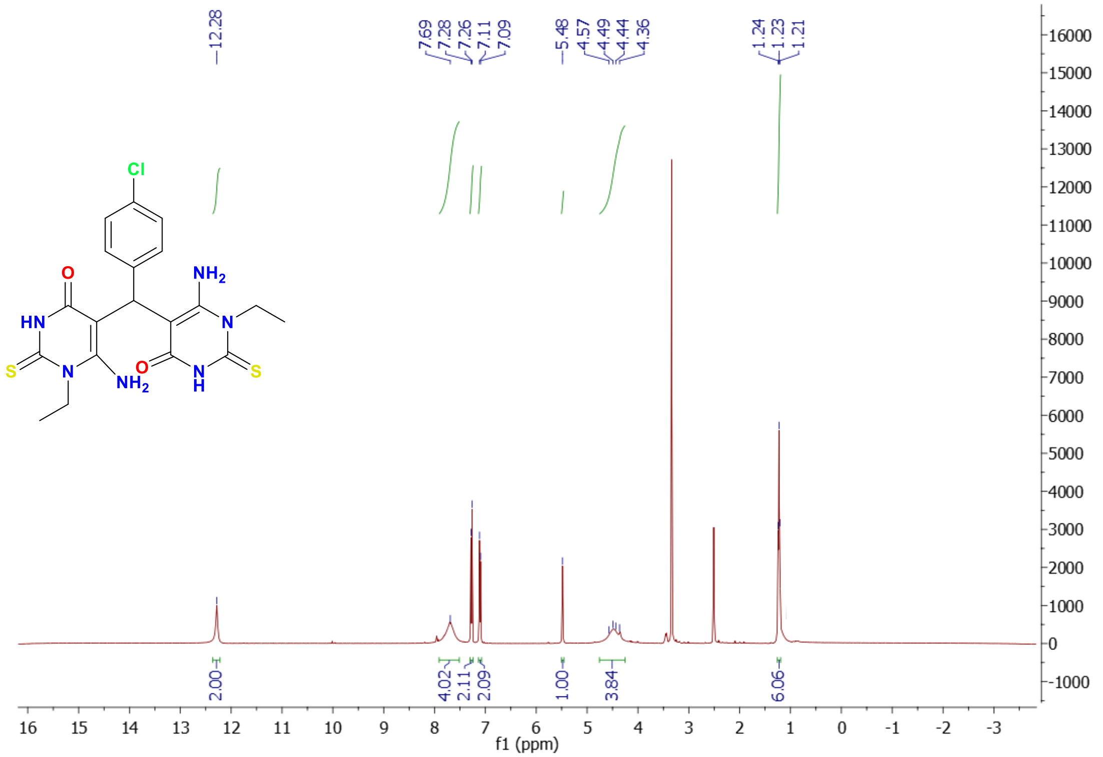

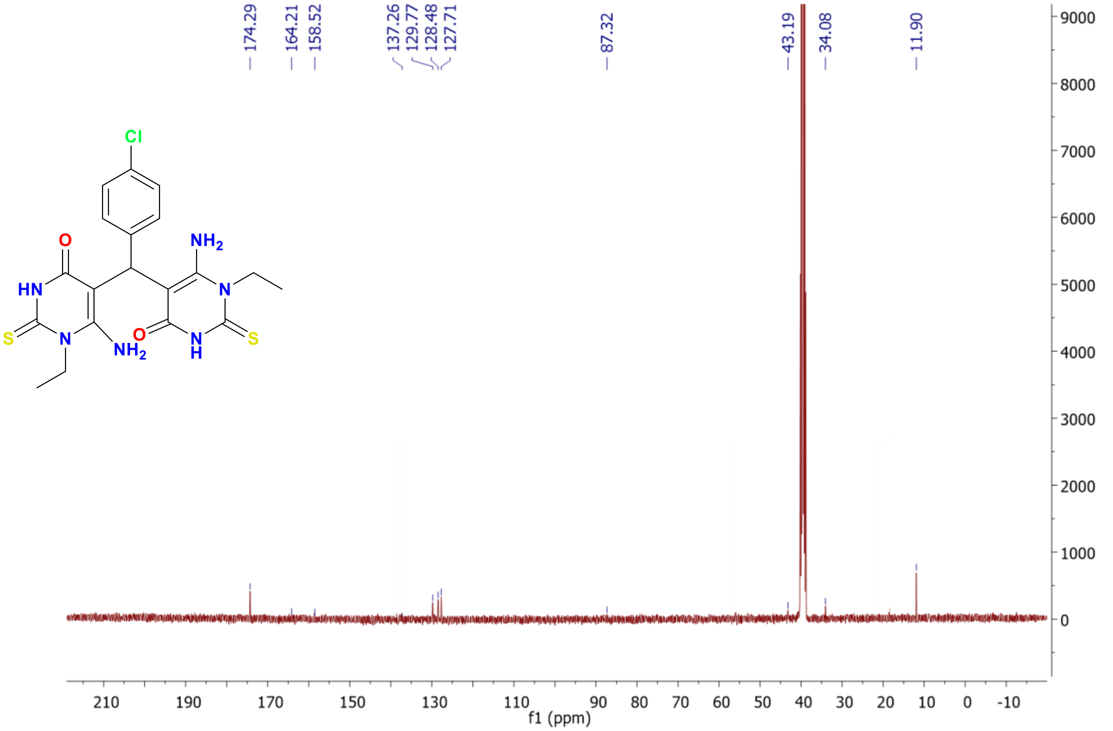

5,5'-((2,4-dichlorophenyl)methylene)bis(6-amino-1-ethyl-2-thioxo-2,3-dihydropyrimidin-4(1*H*)-one) (**5d**)

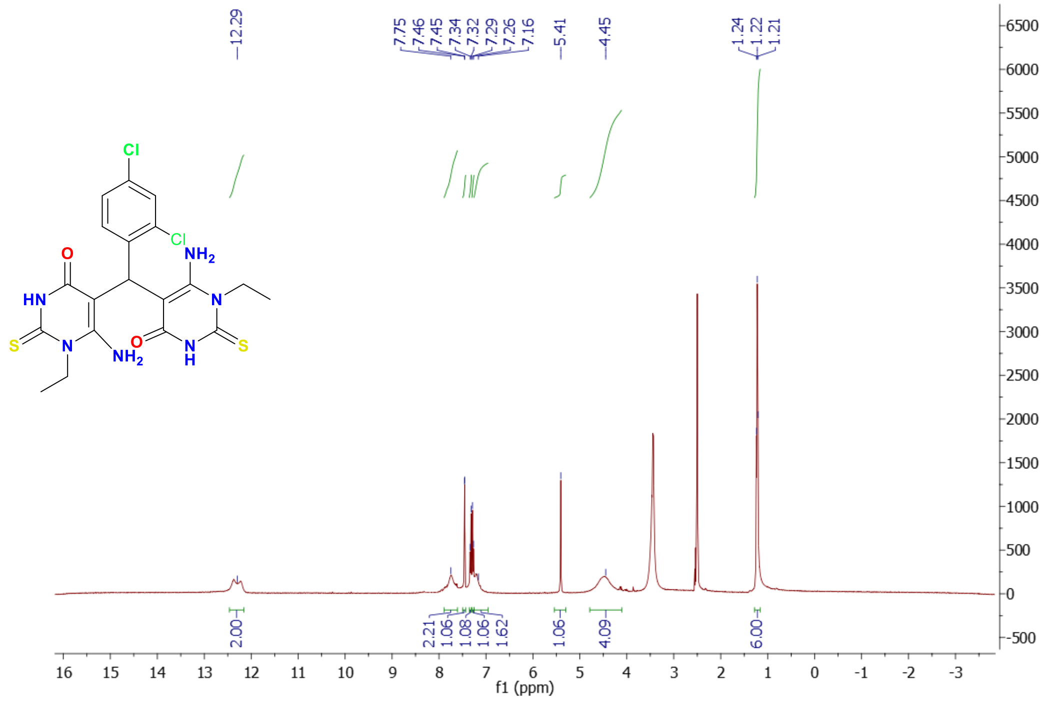

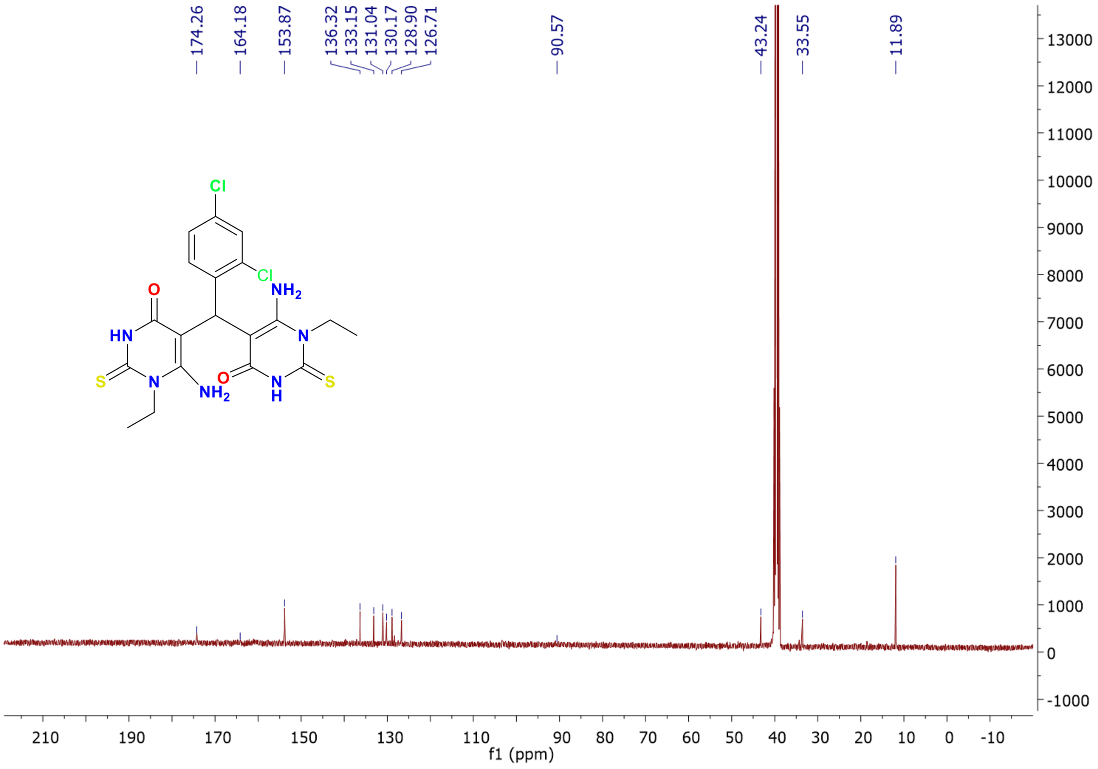

5,5'-((4-methoxyphenyl)methylene)bis(6-amino-1-ethyl-2-thioxo-2,3-dihydropyrimidin-4(1*H*)-one) (**5e)**

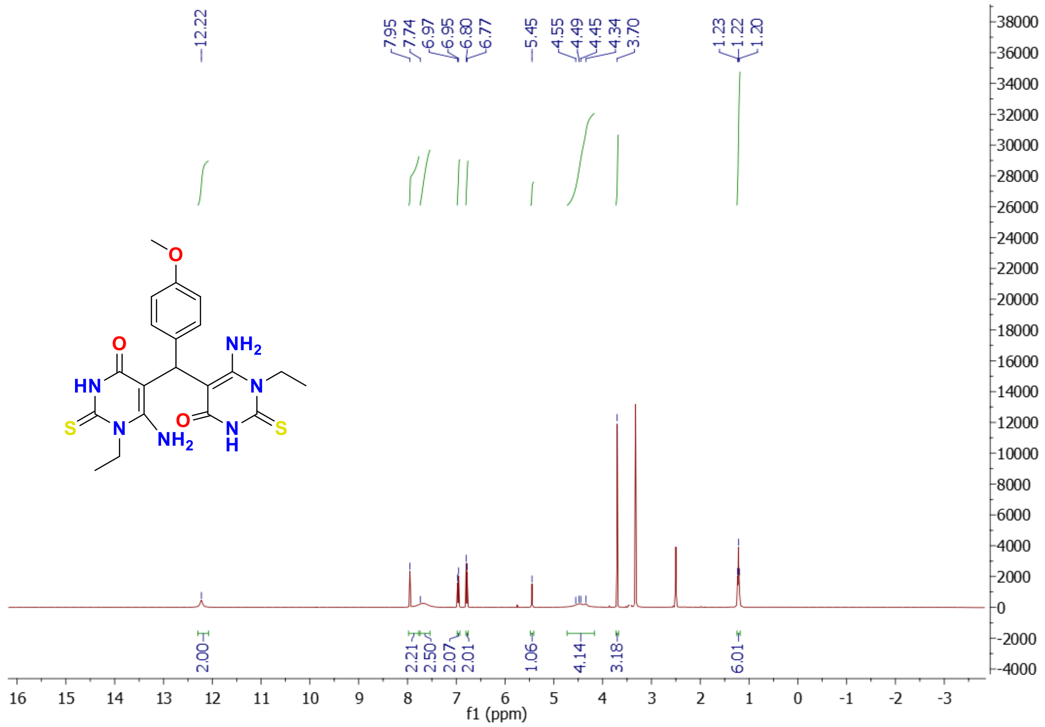

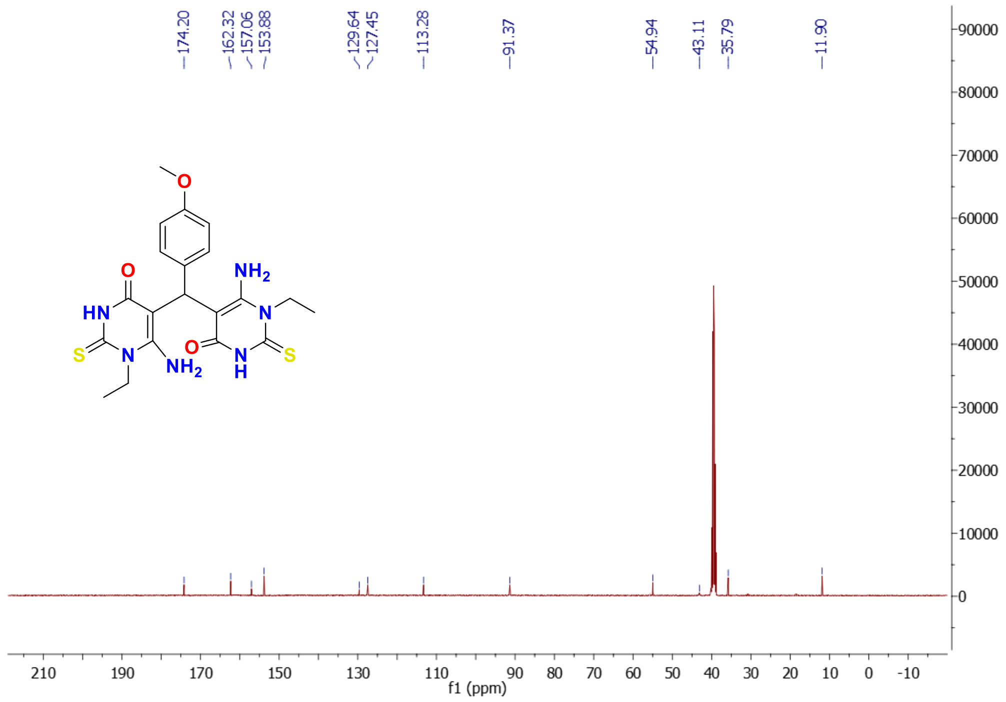

5,5'-((4-nitrophenyl)methylene)bis(6-amino-1-ethyl-2-thioxo-2,3-dihydropyrimidin-4(1*H*)-one) **(5f)**

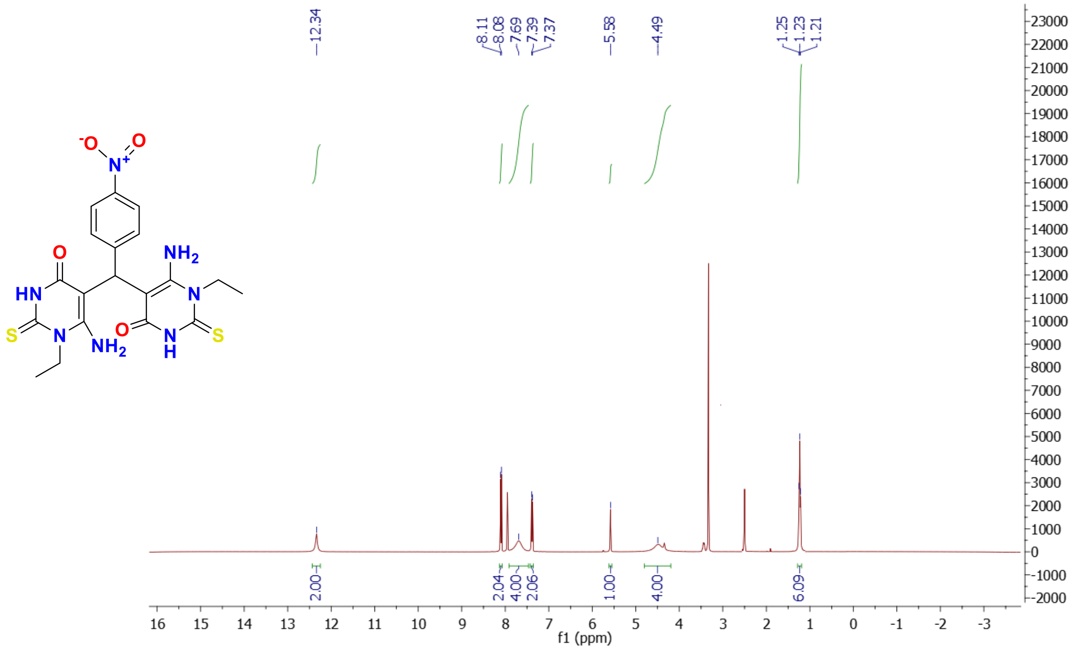

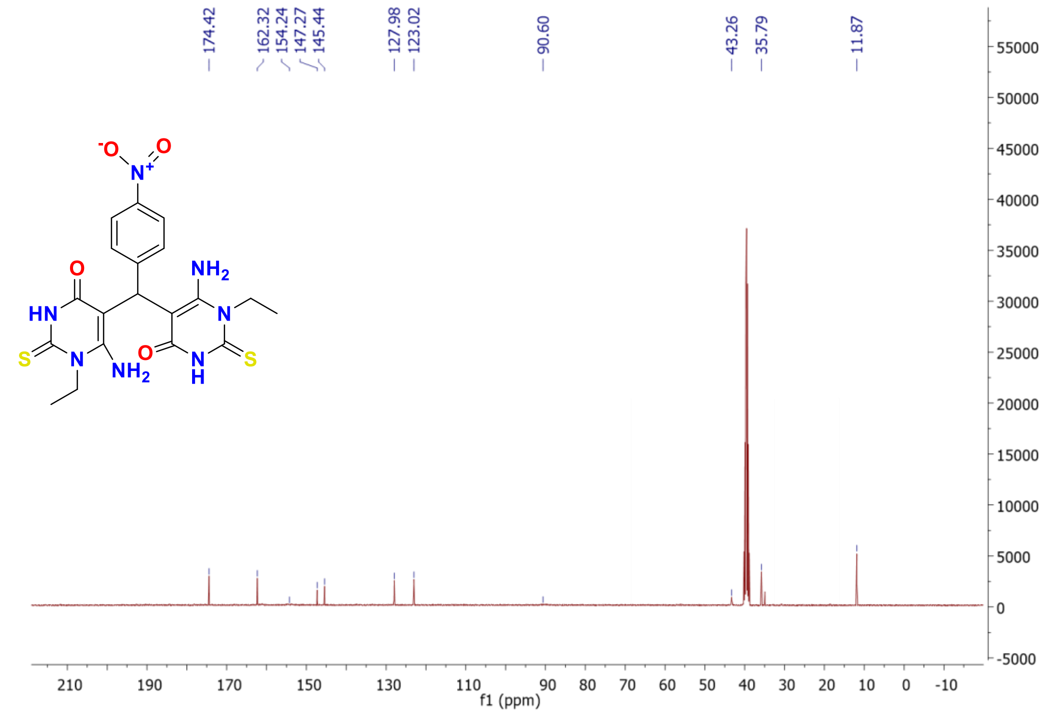

5,5'-(thiophen-2-ylmethylene)bis(6-amino-1-ethyl-2-thioxo-2,3-dihydropyrimidin-4(1*H*)-one) **(5g)**

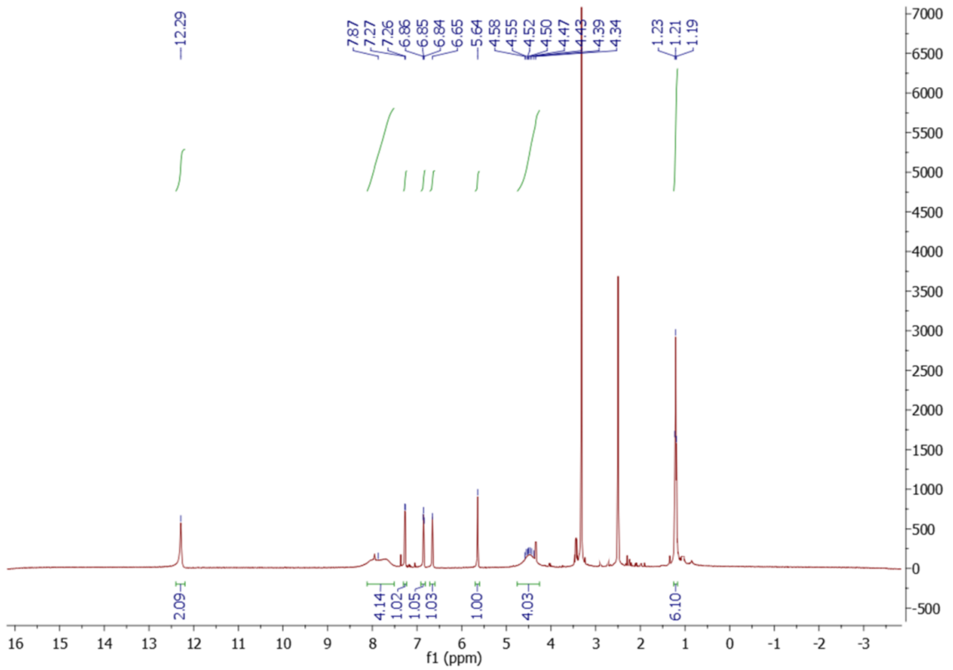

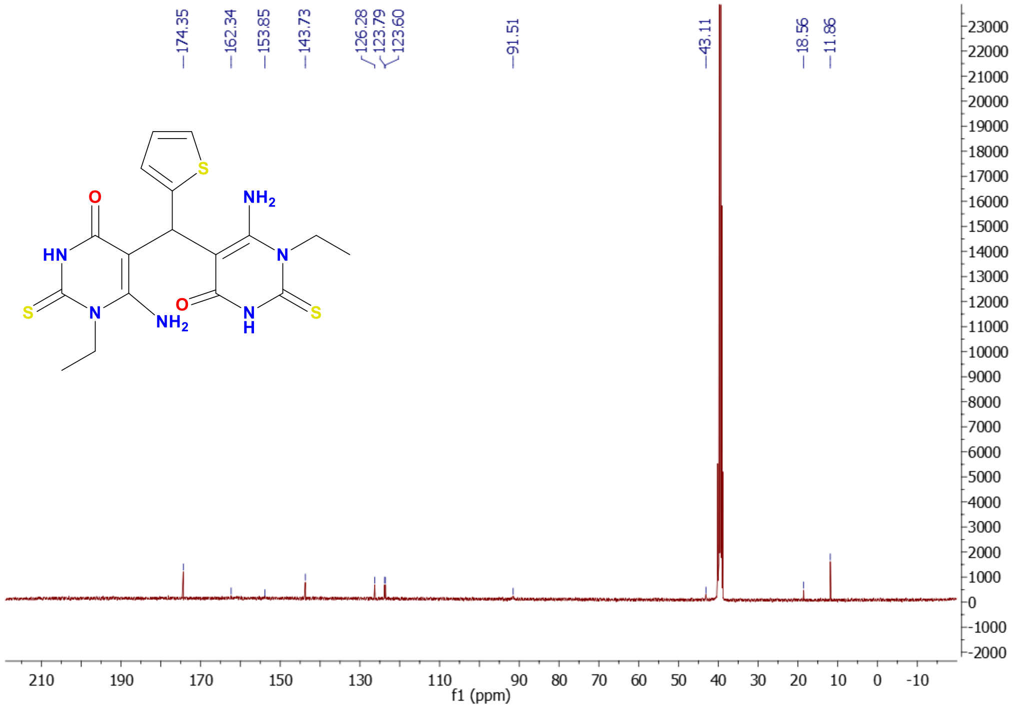

5,5'-methylenebis(6-amino-1-propylpyrimidine-2,4(1*H*,3*H*)-dione) (**5h)**

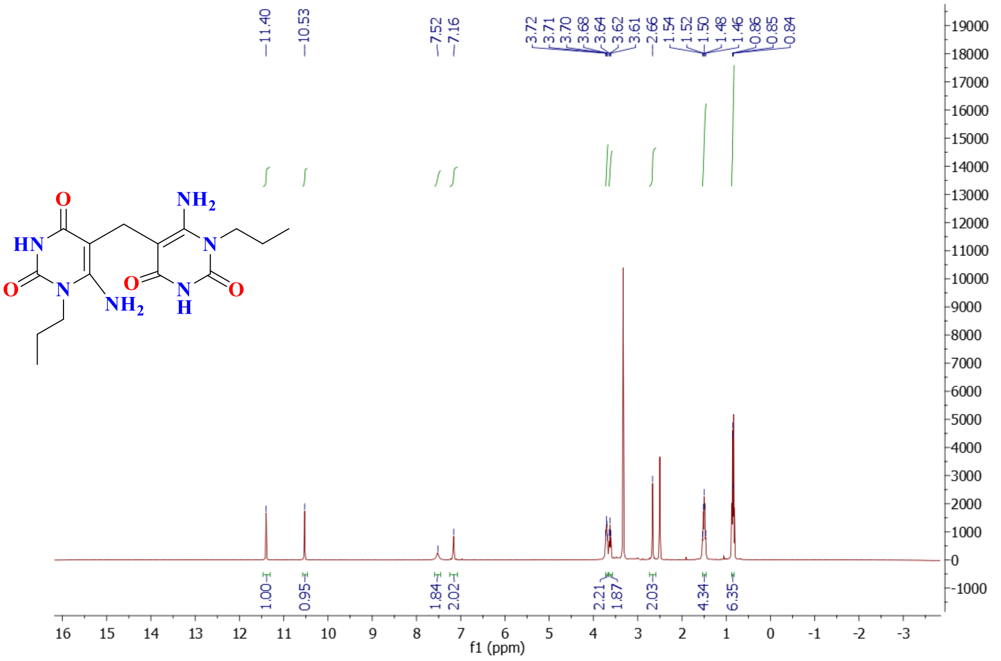

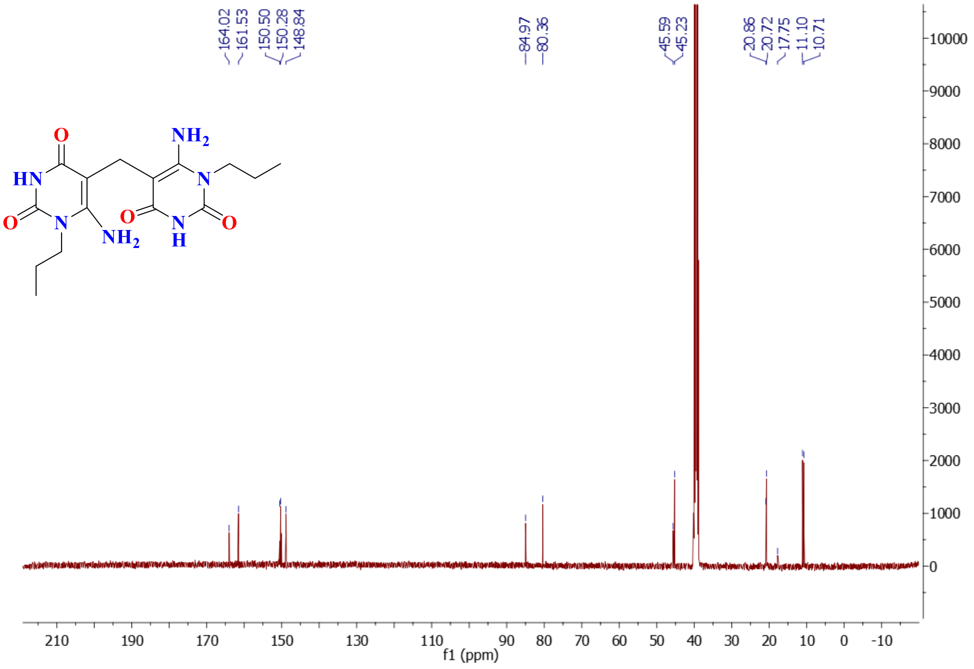

5,5'-(phenylmethylene)bis(6-amino-1-propylpyrimidine-2,4(1*H*,3*H*)-dione) **(5i)**

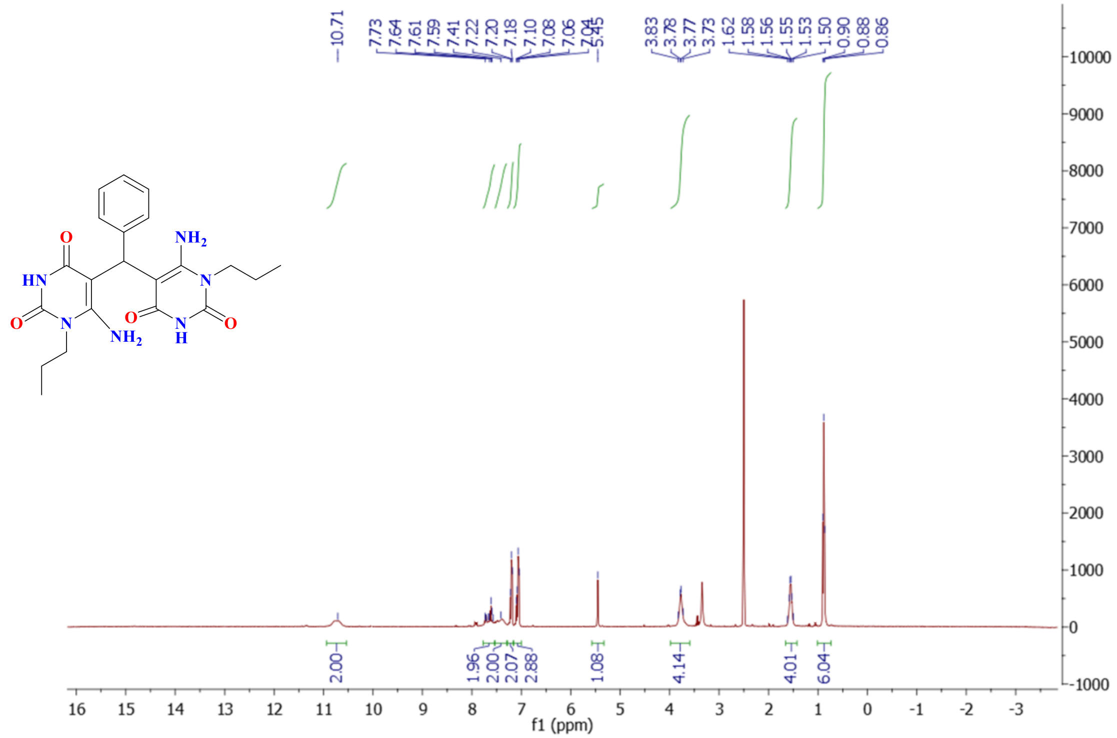

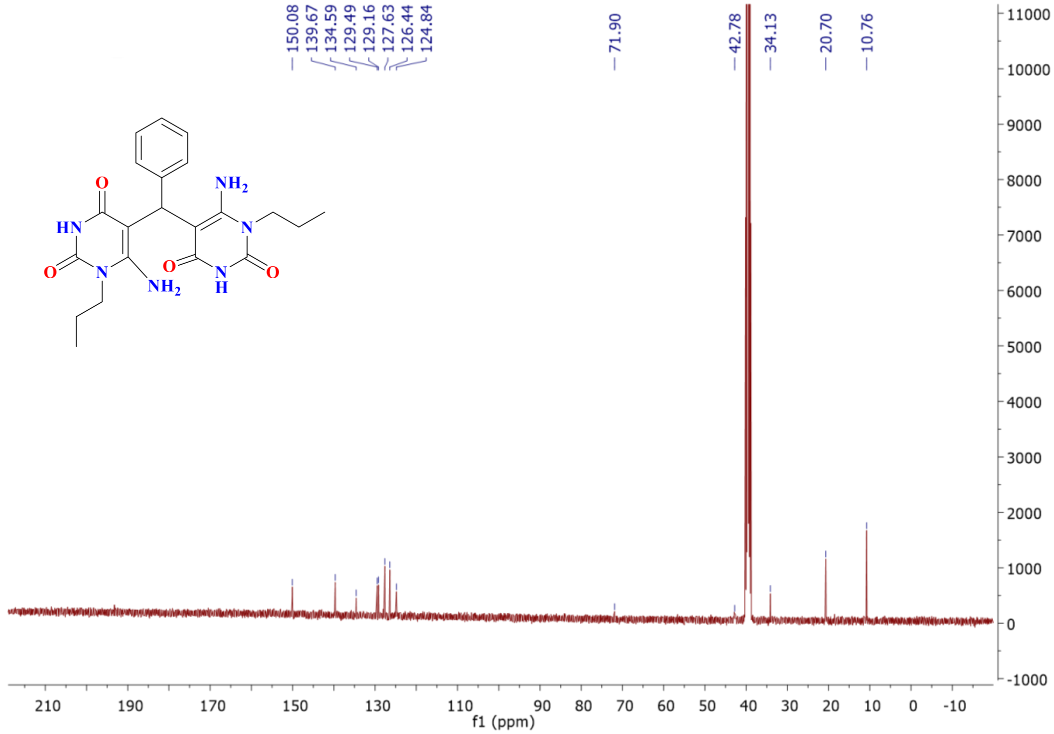

5,5'-(p-tolylmethylene)bis(6-amino-1-propylpyrimidine-2,4(1*H*,3*H*)-dione) **(5j)**

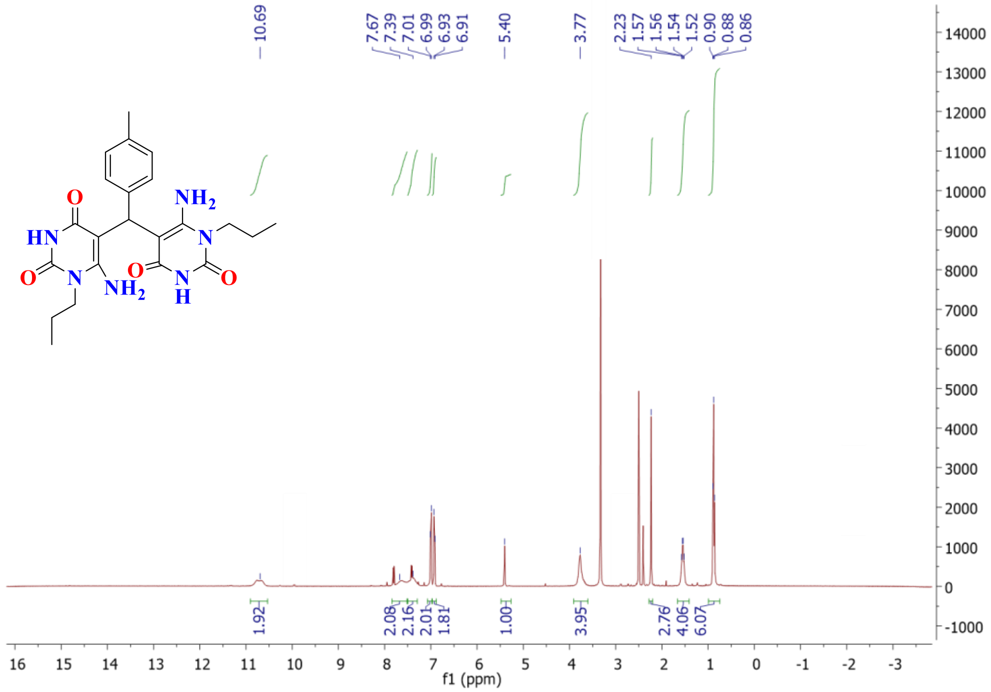

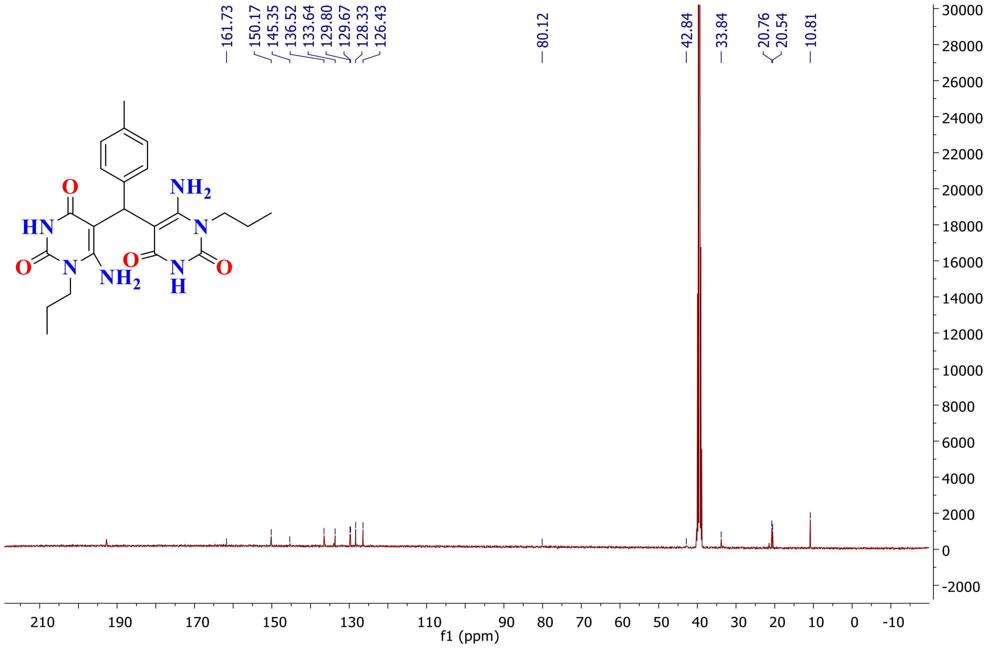

5,5'-((2,4-dichlorophenyl)methylene)bis(6-amino-1-propylpyrimidine-2,4(1*H*,3*H*)-dione) **(5k)**

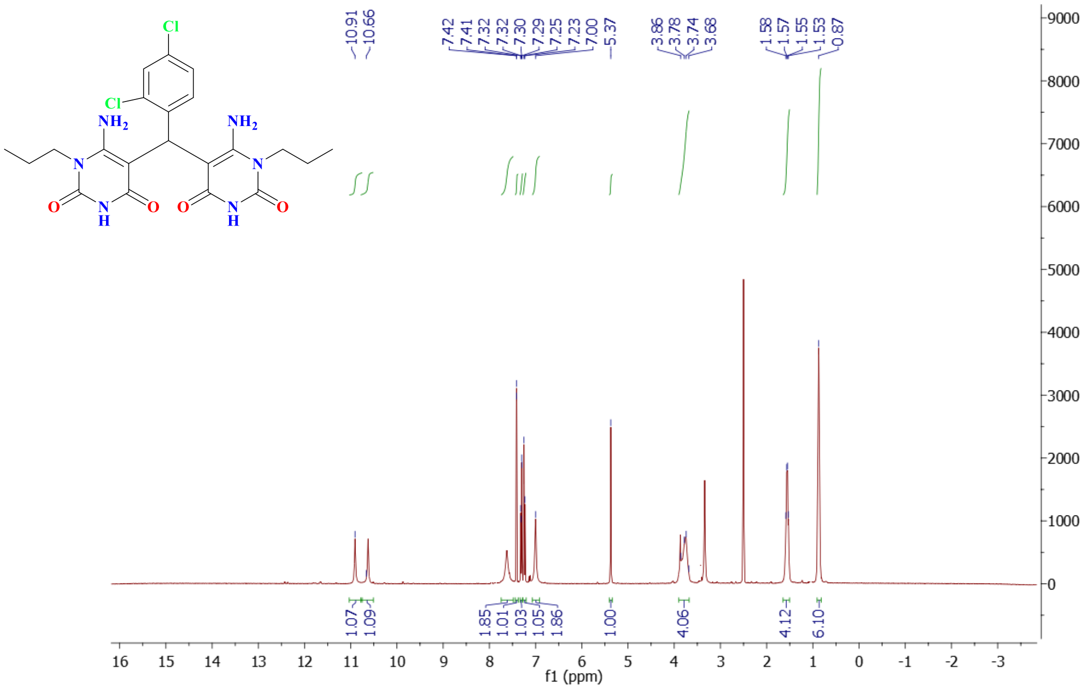

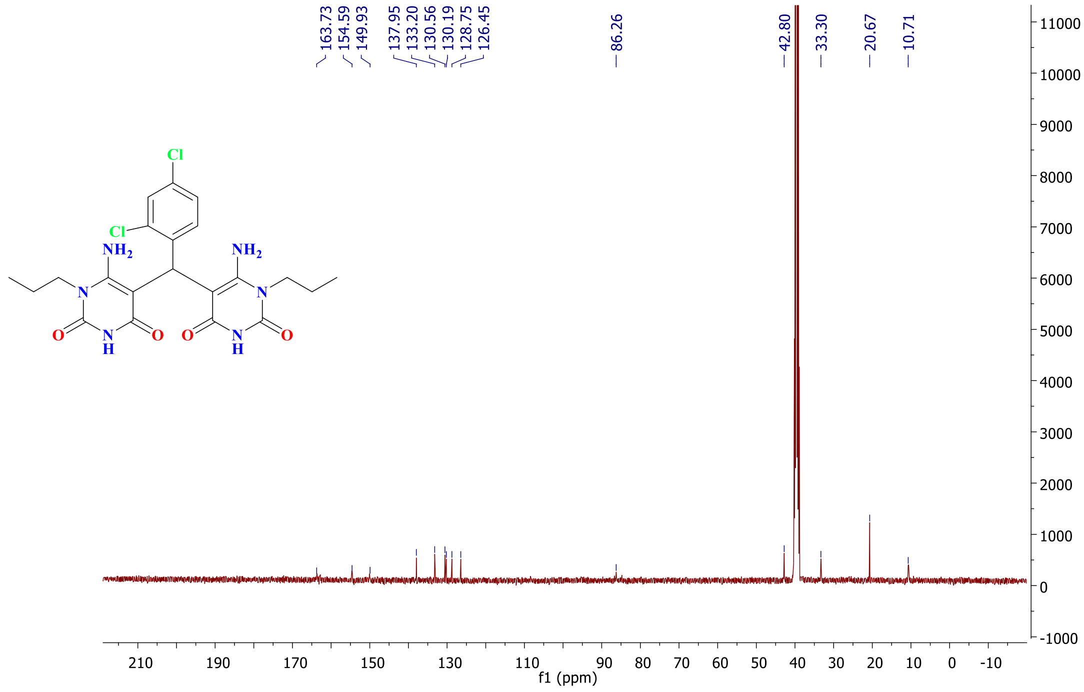

(5,5'-((4-methoxyphenyl)methylene)bis(6-amino-1-propylpyrimidine-2,4(1*H*,3*H*)-dione) **(5L)**

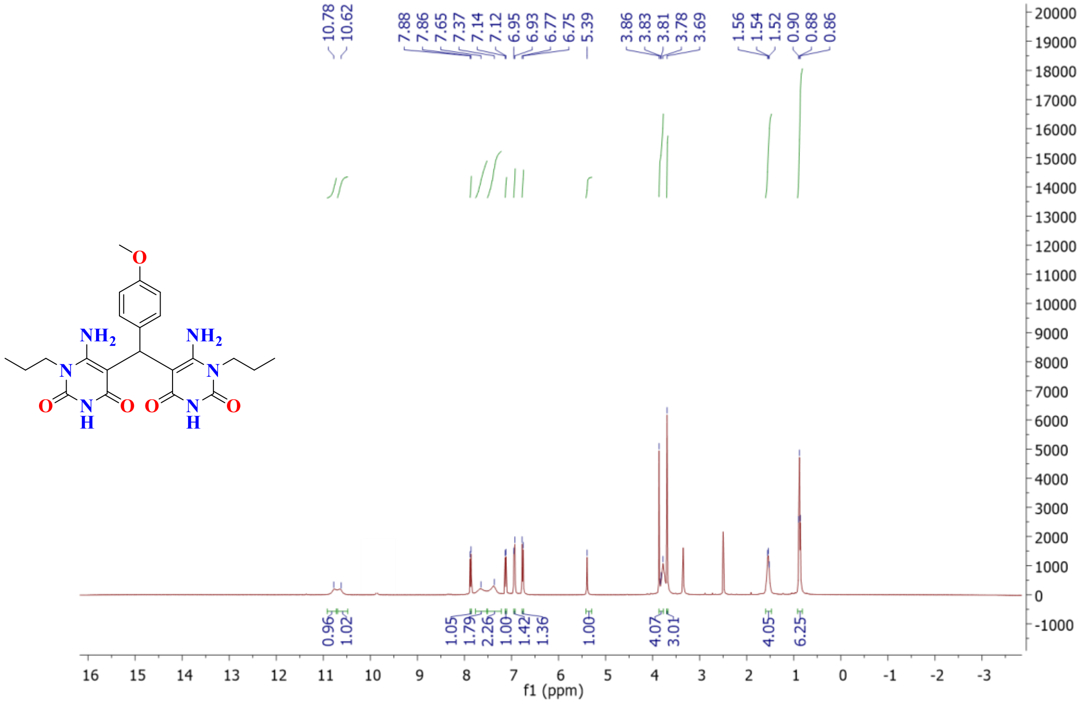

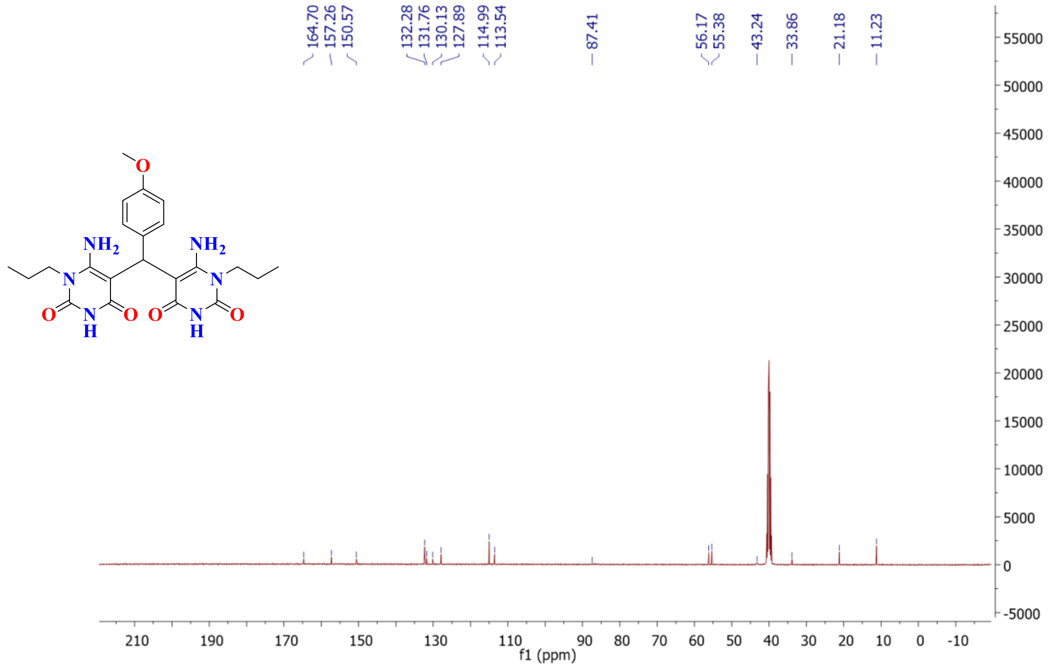

5,5'-((4-chlorophenyl)methylene)bis(6-amino-1-propylpyrimidine-2,4(1*H*,3*H*)-dione) (**5m)**

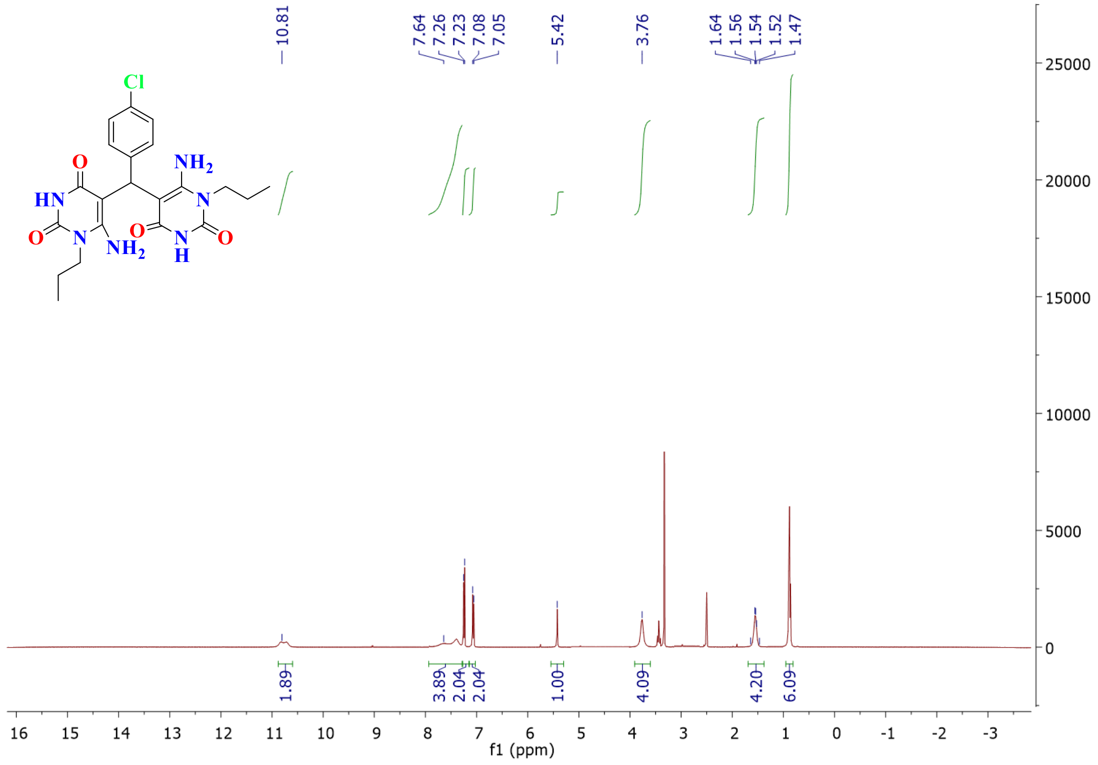

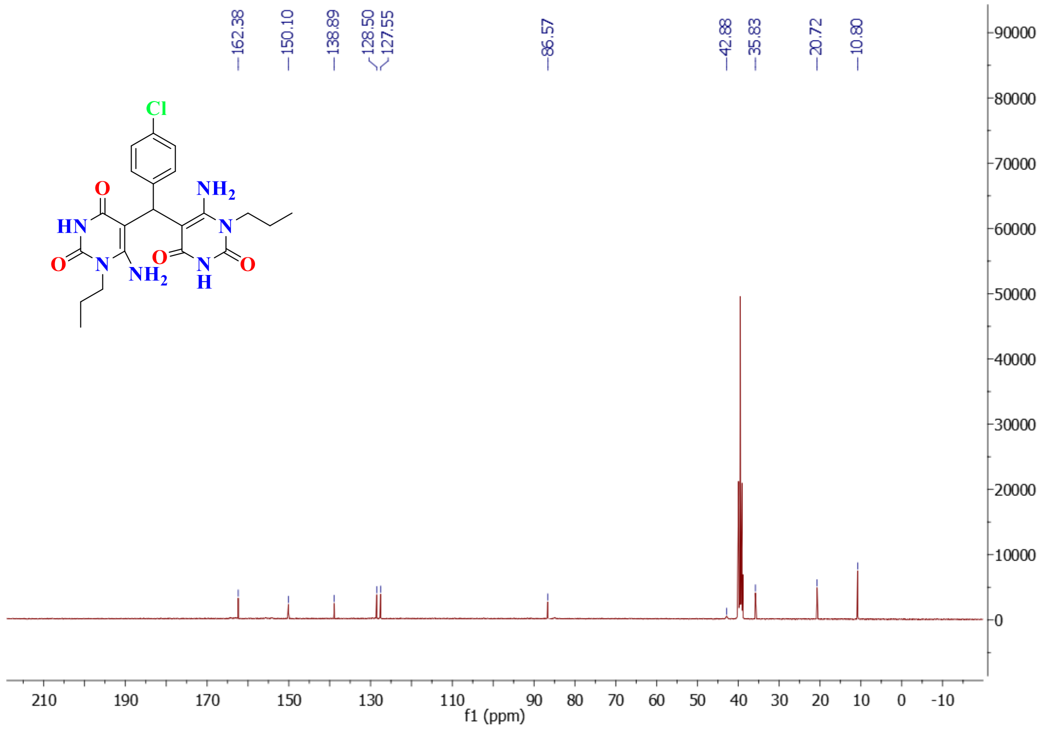

1,9-diethyl-5-phenyl-2,8-dithioxo-2,3,5,8,9,10-hexahydropyrido[2,3-d:6,5-d']dipyrimidine-4,6(1*H*,7*H*)-dione **(6a)**

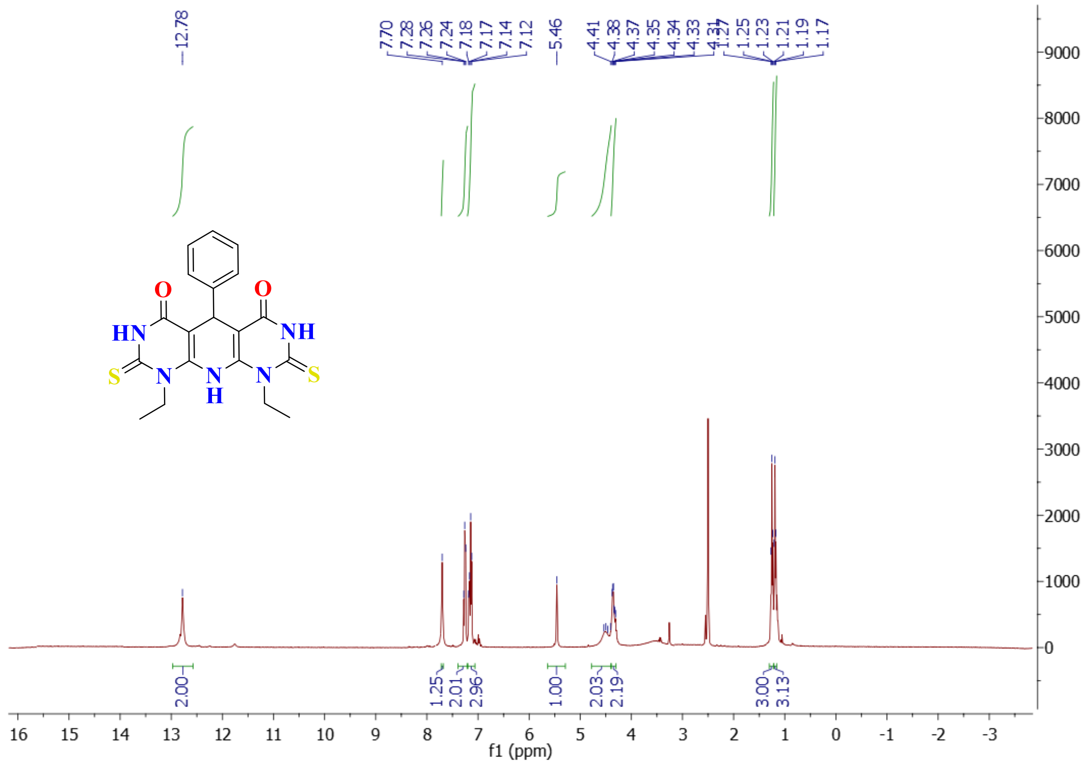

1,9-diethyl-5-(4-nitrophenyl)-2,8-dithioxo-2,3,5,8,9,10-hexahydropyrido[2,3-d:6,5-d'] dipyrimidine-4,6(1*H*,7*H*)-dione **(6b)**

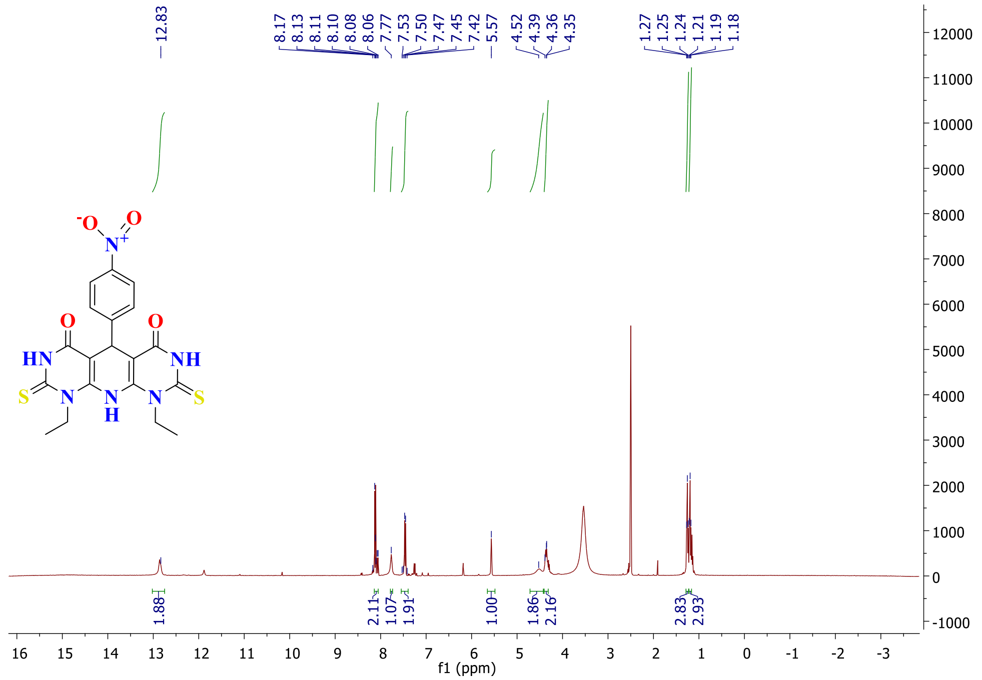

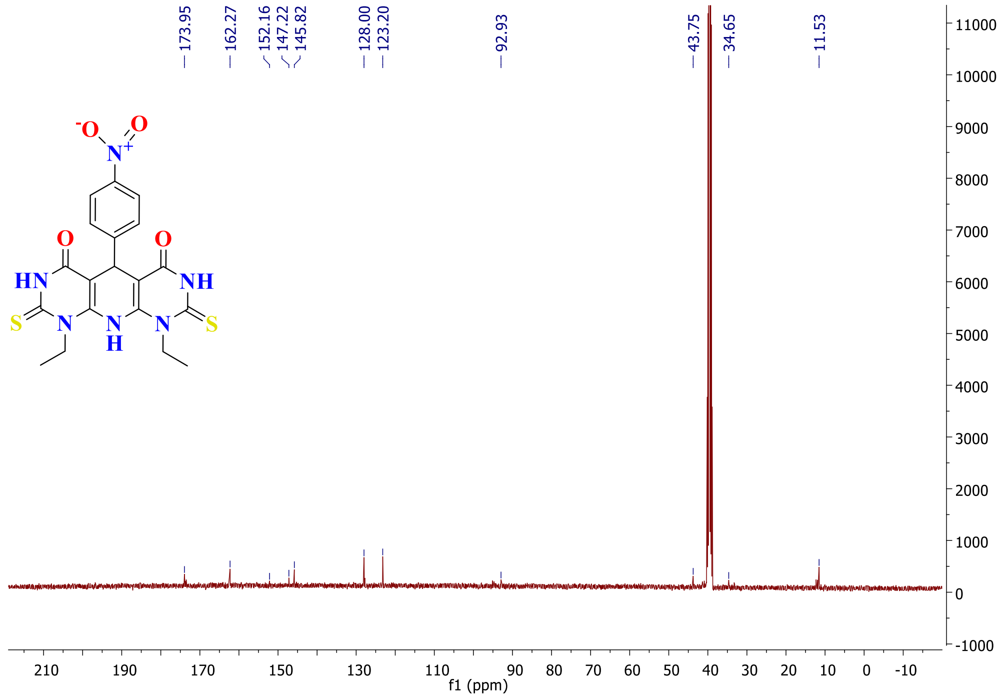

5-(4-methoxyphenyl)-1,9-dipropyl-5,10-dihydropyrido[2,3-d:6,5-d']dipyrimidine-2,4,6,8(1*H*,3*H*,7*H*,9*H*)-tetraone **(6c)**

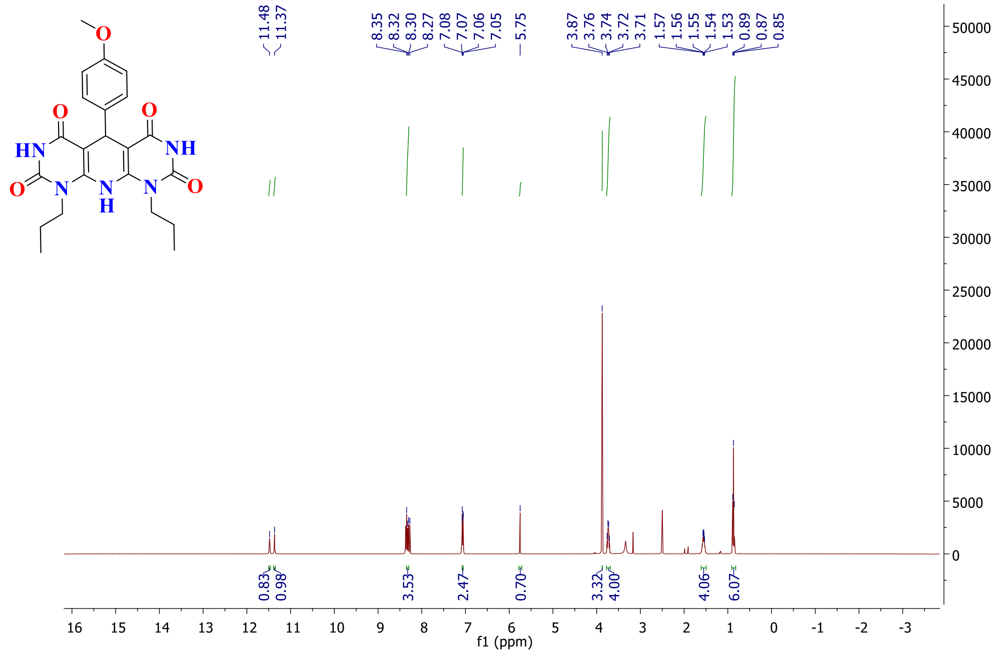

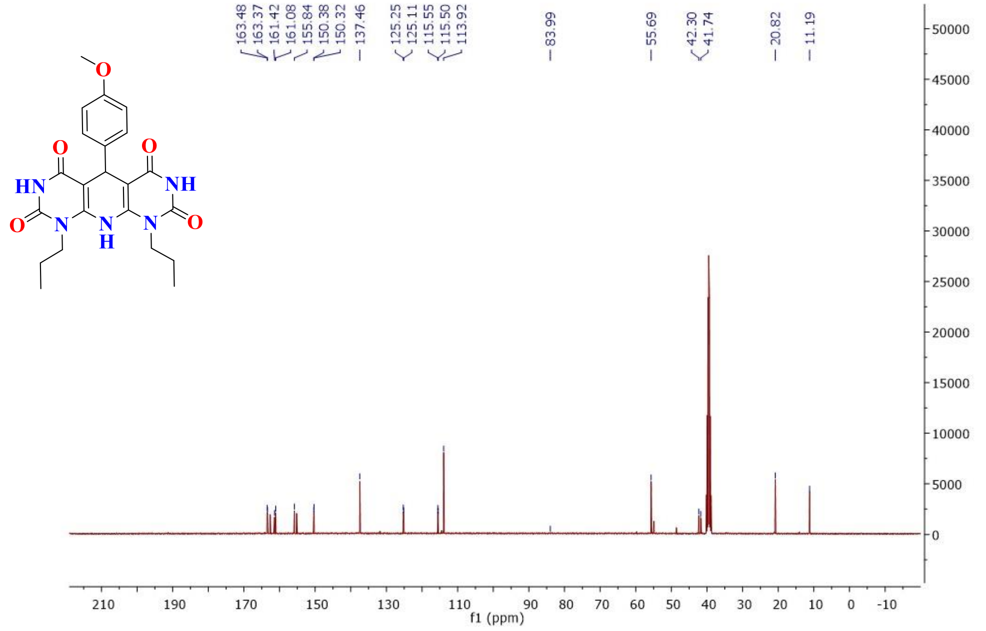

1,9-dipropyl-5,10-dihydropyrido[2,3-d:6,5-d']dipyrimidine-2,4,6,8(1H,3H,7H,9H)-tetraone **(6d)**

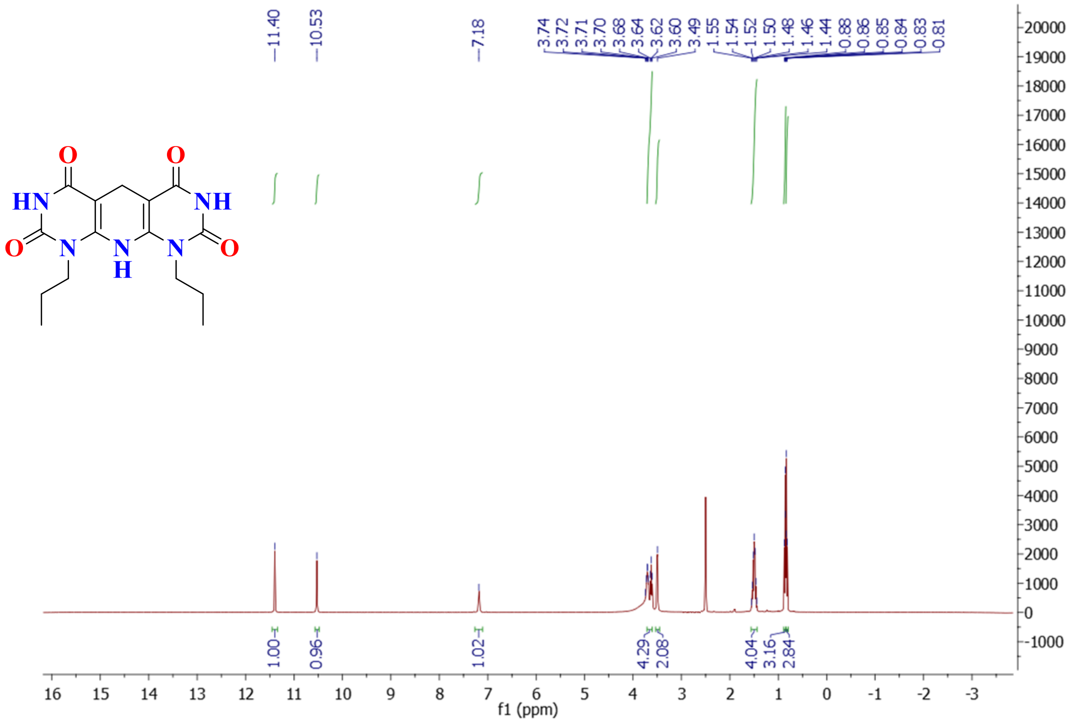

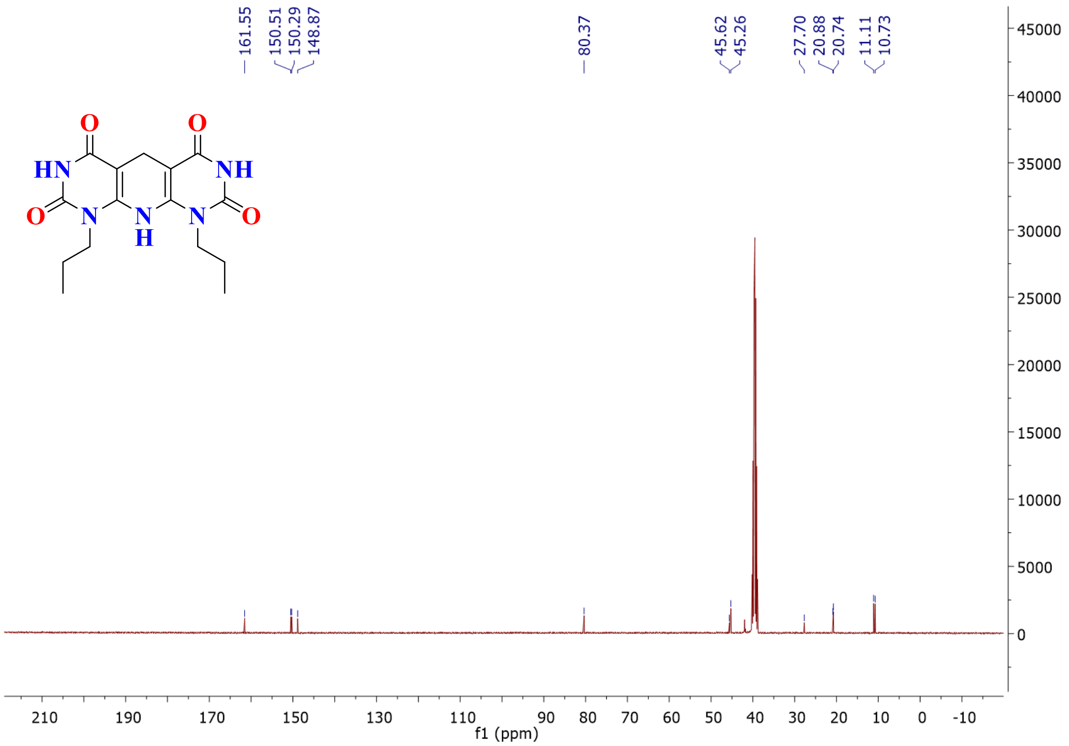

5-(4-chlorophenyl)-1,9-diethyl-2,8-dithioxo-2,3,8,9-tetrahydropyrido[2,3-d:6,5-d']dipyrimidine-4,6(1H,7*H*)-dione **(7a)**

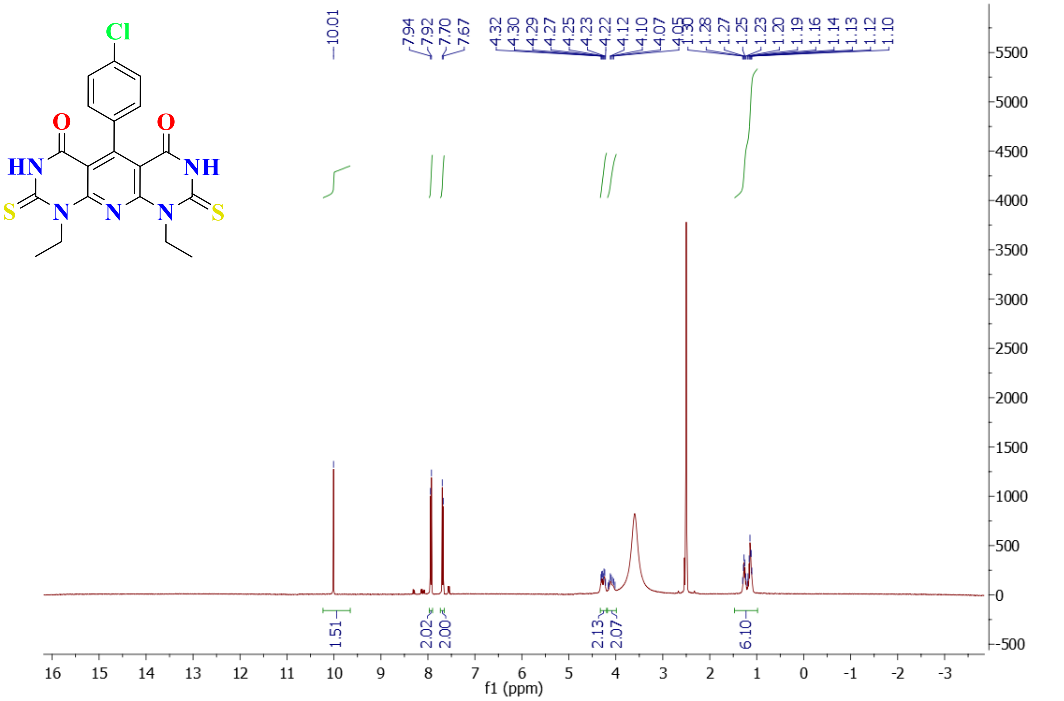

1,9-diethyl-5-(4-methoxyphenyl)-2,8-dithioxo-2,3,8,9-tetrahydropyrido[2,3-d:6,5-d']dipyrimidine-4,6(1*H*,7*H*)-dione **(7b)**

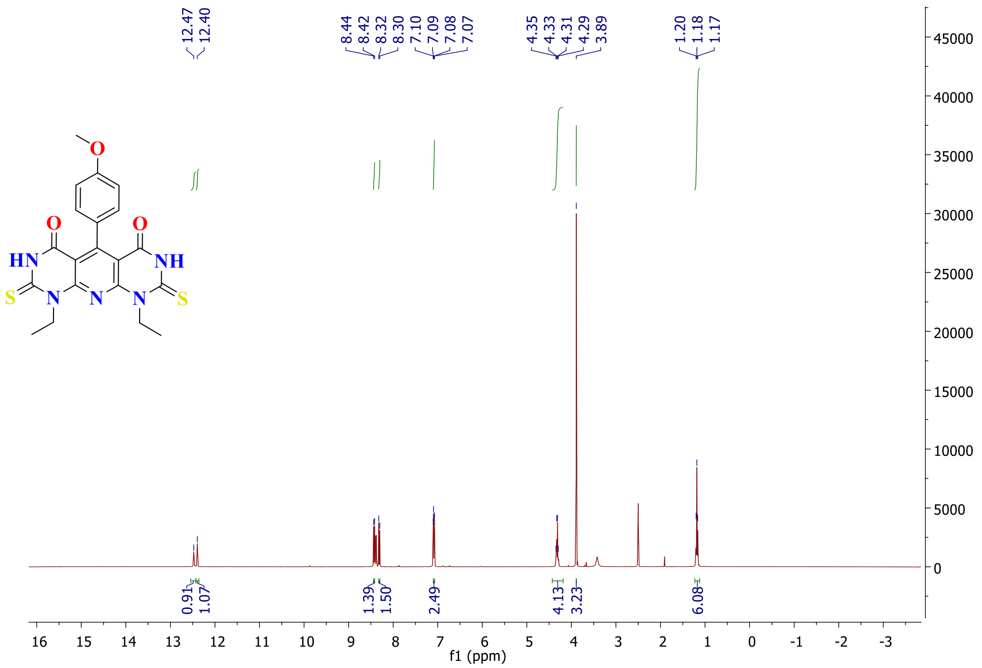

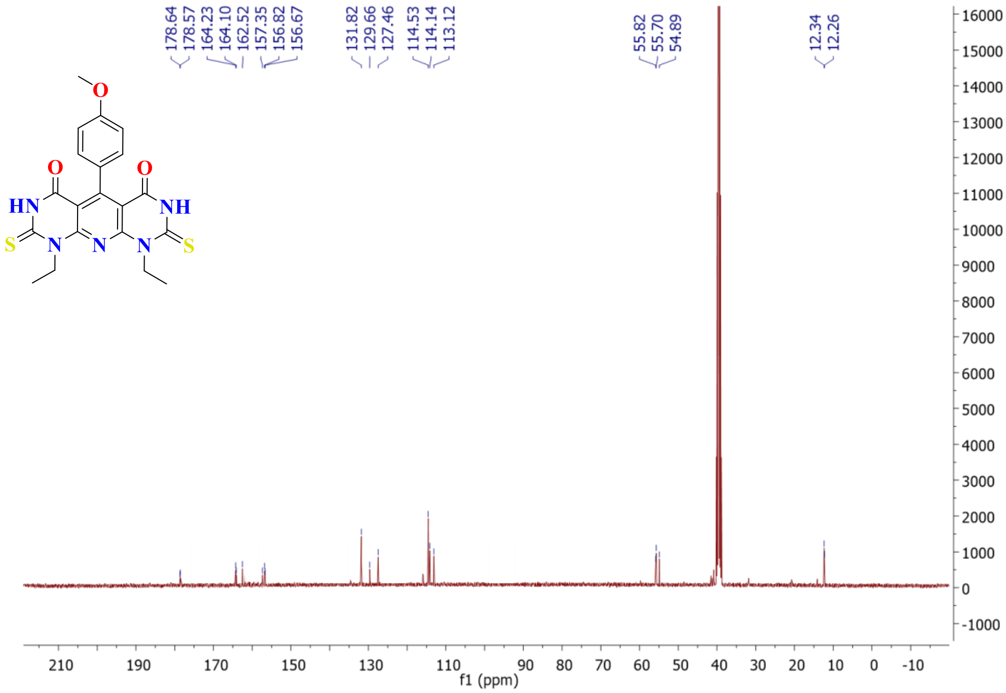

1,9-diethyl-5-(thiophen-2-yl)-2,8-dithioxo-2,3,8,9-tetrahydropyrido[2,3-d:6,5-d']dipyrimidine-4,6(1H,7H)-dione **(7c)**

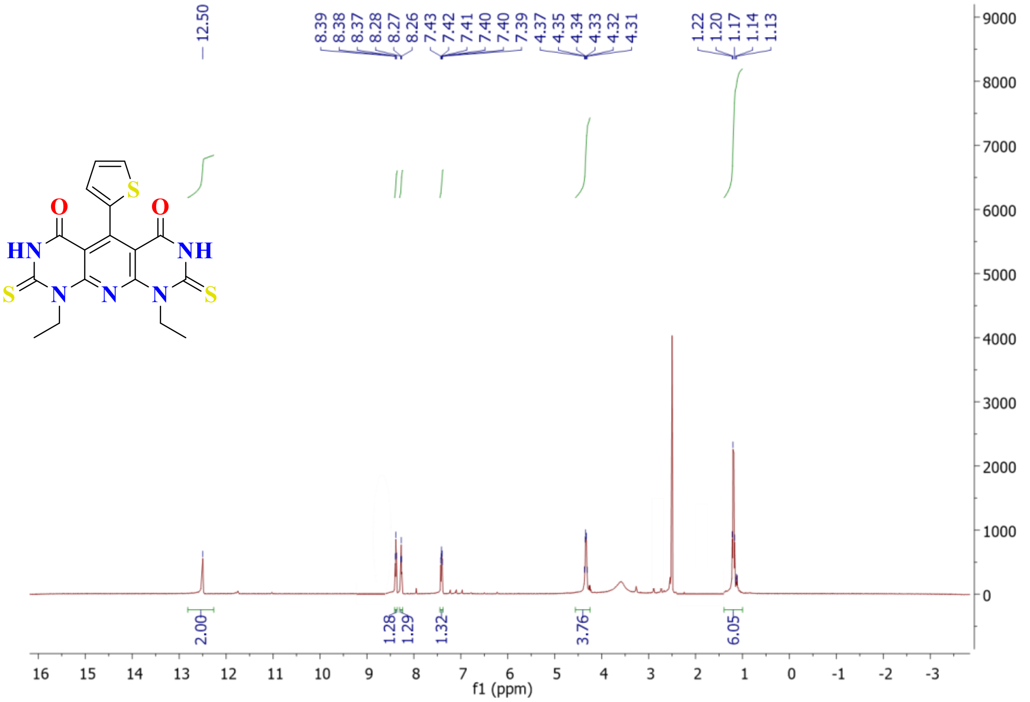

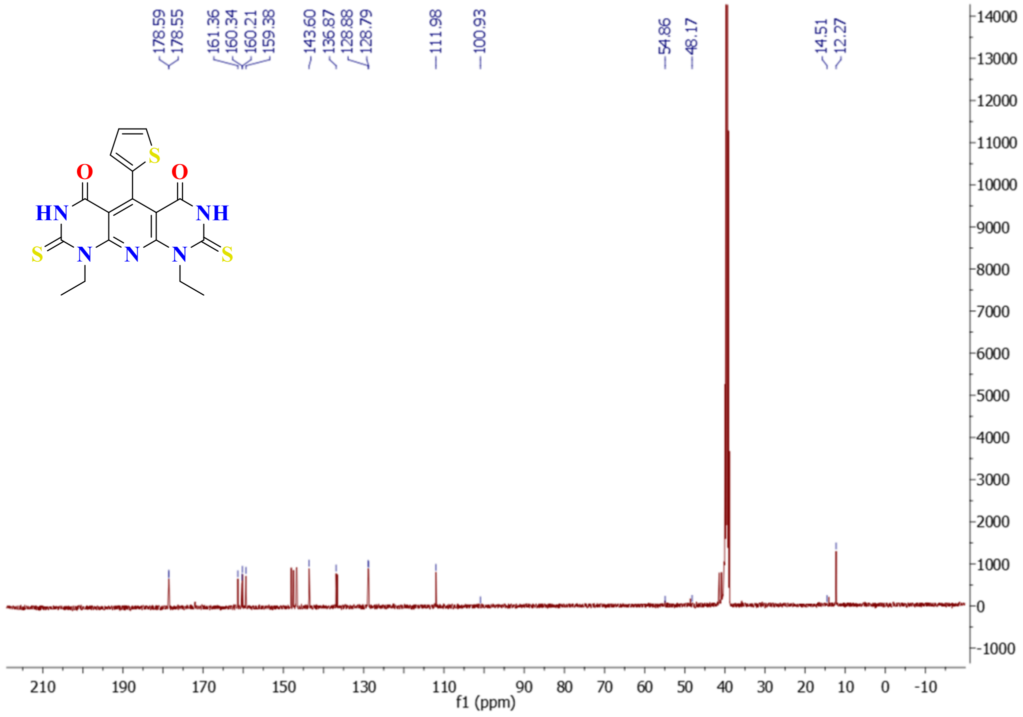

5,5'-(phenylmethylene)bis(6-amino-1-ethyl-2-thioxo-2,3-dihydropyrimidin-4(1*H*)-one) (**5a)**

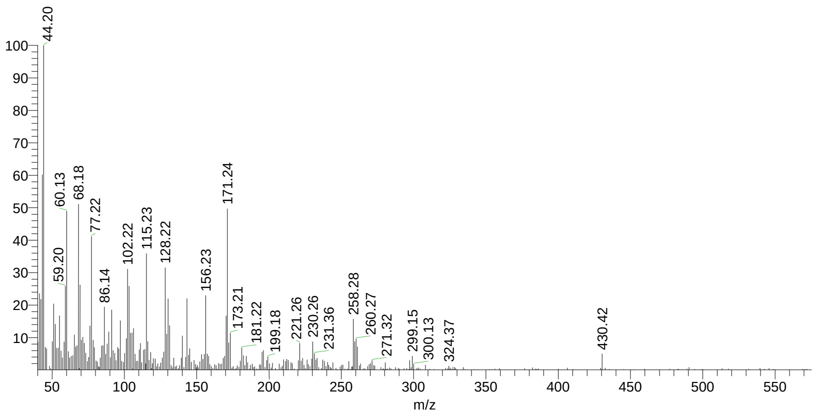

5,5'-(p-tolylmethylene)bis(6-amino-1-ethyl-2-thioxo-2,3-dihydropyrimidin-4(1*H*)-one) (**5b)**

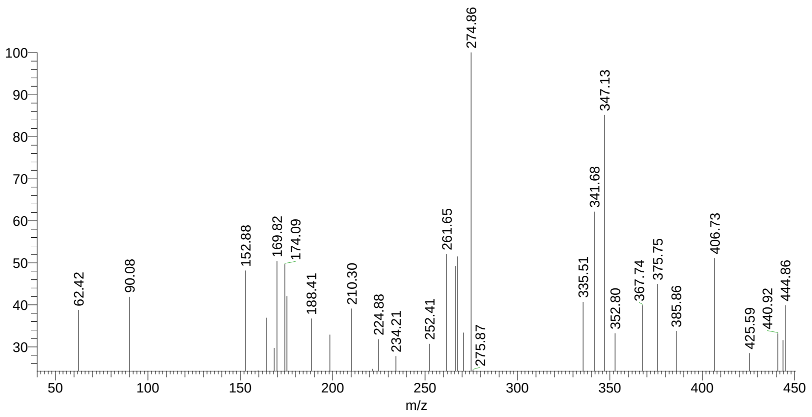

5,5'-((4-chlorophenyl)methylene)bis(6-amino-1-ethyl-2-thioxo-2,3-dihydropyrimidin-4(1*H*)-one) **(5c)**

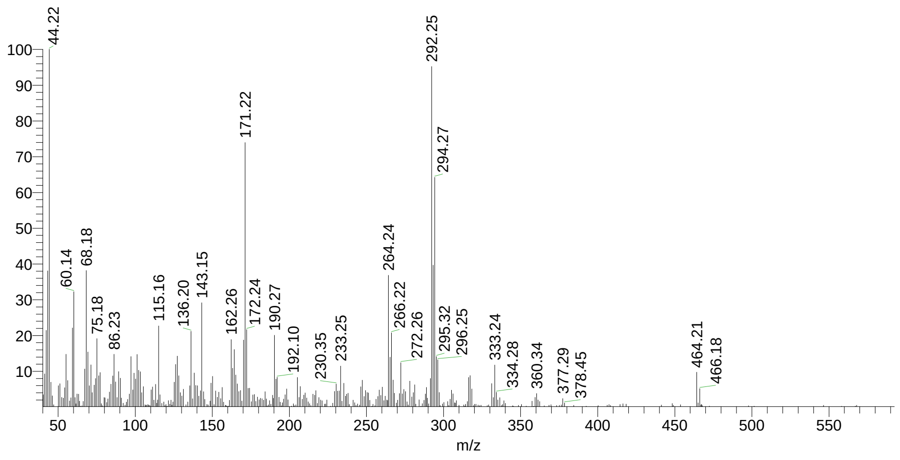

5,5'-((3,4-dichlorophenyl)methylene)bis(6-amino-1-ethyl-2-thioxo-2,3-dihydropyrimidin-4(1*H*)-one) **(5d)**

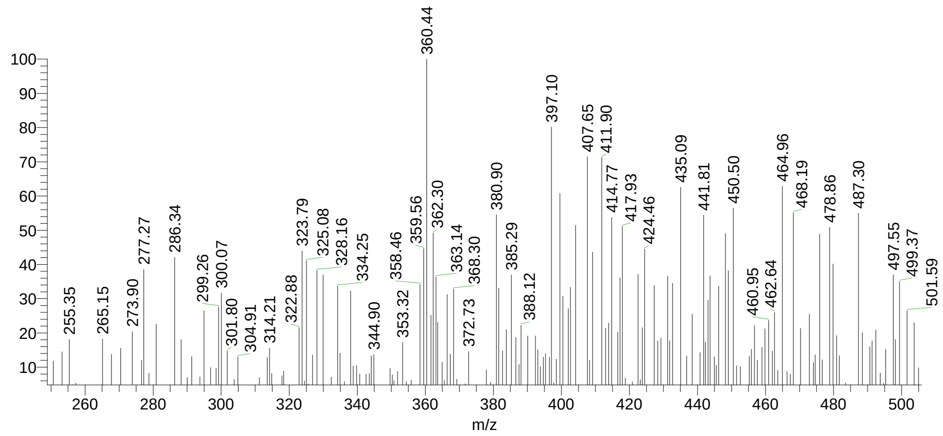

5,5'-((4-methoxyphenyl)methylene)bis(6-amino-1-ethyl-2-thioxo-2,3-dihydropyrimidin-4(1*H*)-one) **(5e)**

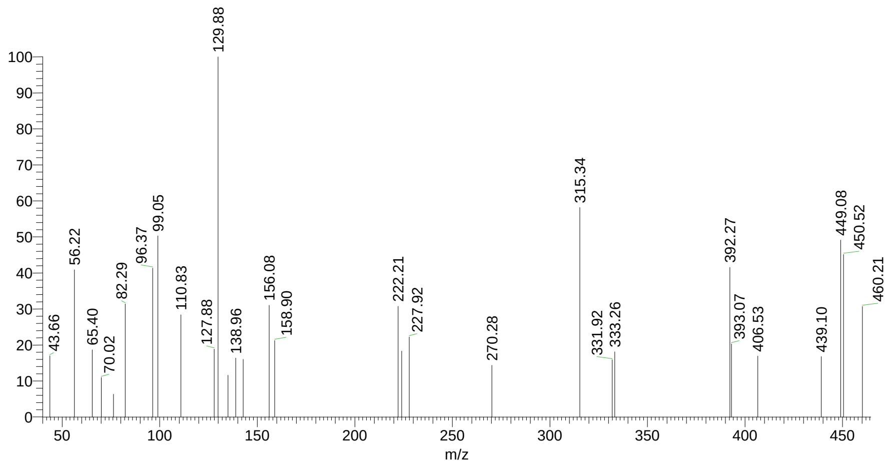

5,5'-((4-nitrophenyl)methylene)bis(6-amino-1-ethyl-2-thioxo-2,3-dihydropyrimidin-4(1*H*)-one) **(5f)**

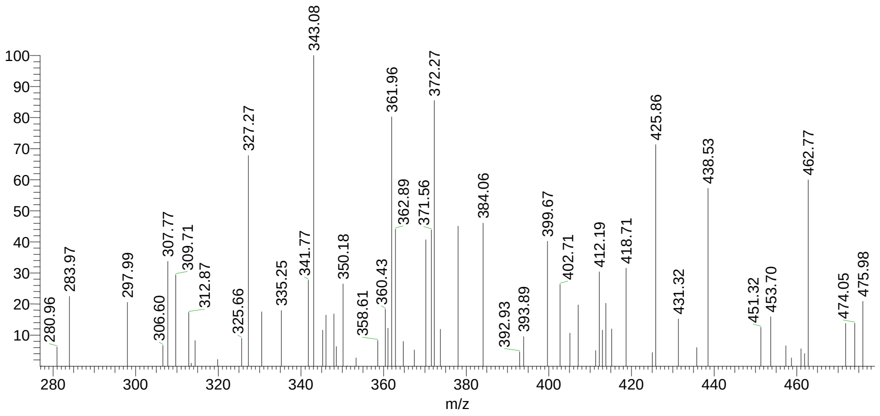

5,5'-(thiophen-2-ylmethylene)bis(6-amino-1-ethyl-2-thioxo-2,3-dihydropyrimidin-4(1*H*)-one) **(5g)**

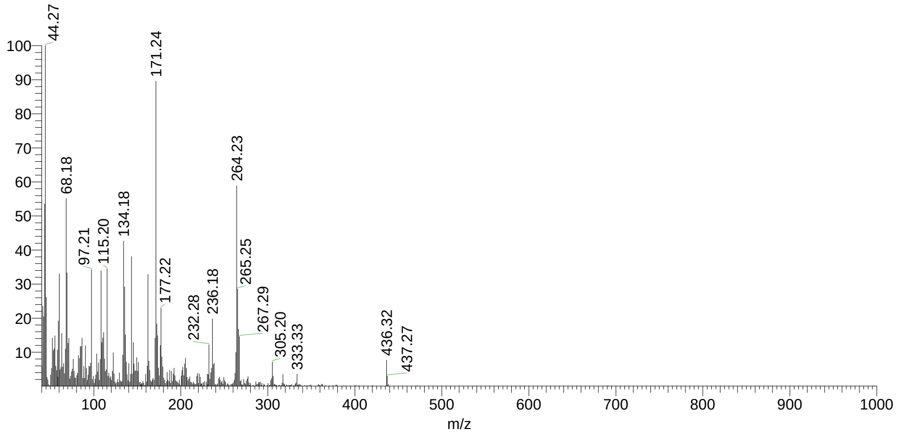

5,5'-methylenebis(6-amino-1-propylpyrimidine-2,4(1*H*,3*H*)-dione) **(5h)**

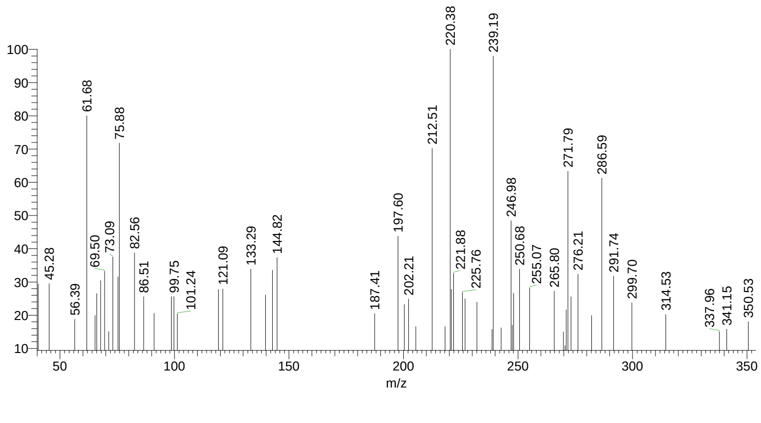

5,5'-(phenylmethylene)bis(6-amino-1-propylpyrimidine-2,4(1*H*,3*H*)-dione) **(5i)**

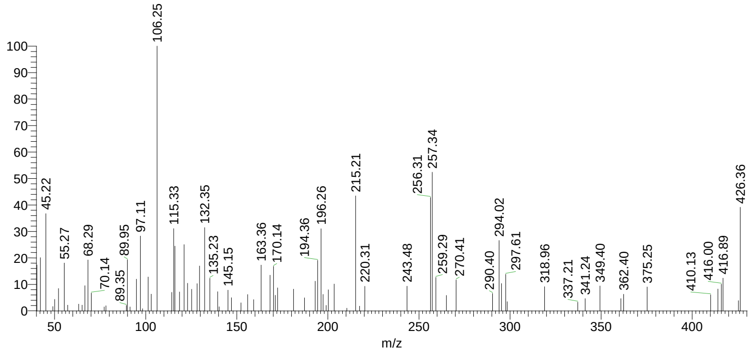

5,5'-(p-tolylmethylene)bis(6-amino-1-propylpyrimidine-2,4(1*H*,3*H*)-dione) **(5j)**

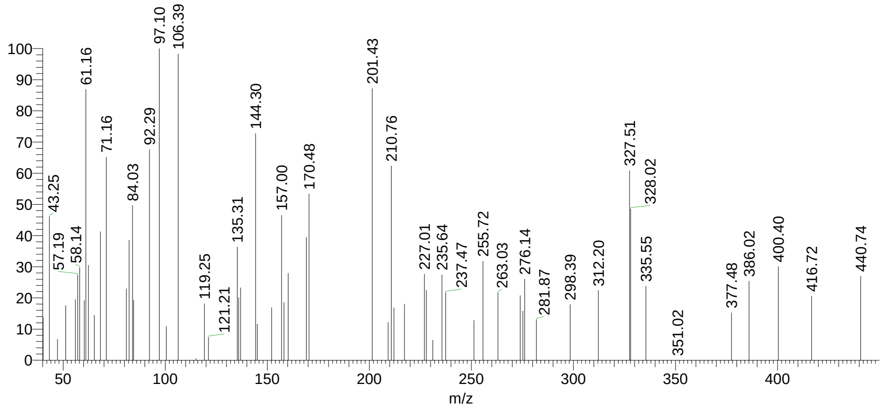

5,5'-((2,4-dichlorophenyl)methylene)bis(6-amino-1-propylpyrimidine-2,4(1*H*,3*H*)-dione) **(5k)**

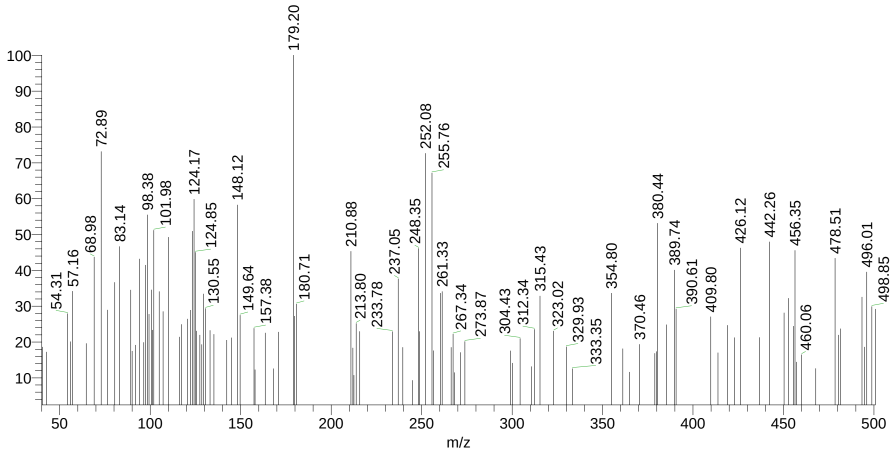

(5,5'-((4-methoxyphenyl)methylene)bis(6-amino-1-propylpyrimidine-2,4(1*H*,3*H*)-dione) **(5l)**

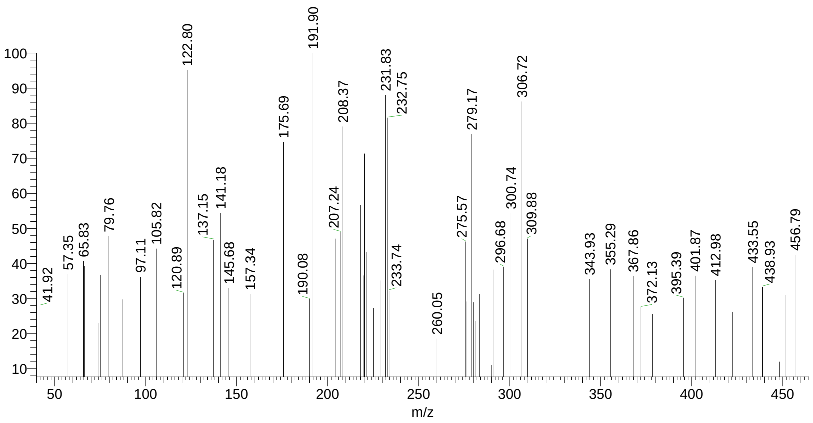

5,5'-((4-chlorophenyl)methylene)bis(6-amino-1-propylpyrimidine-2,4(1*H*,3*H*)-dione) (**5m)**

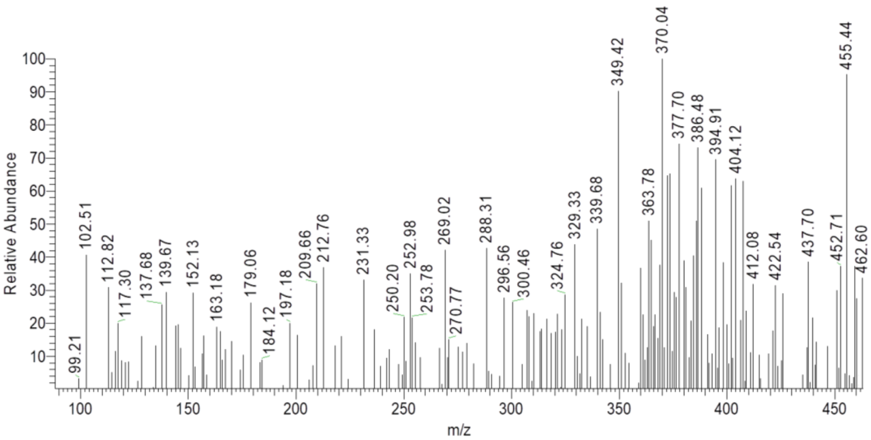

1,9-diethyl-5-phenyl-2,8-dithioxo-2,3,5,8,9,10-hexahydropyrido[2,3-d:6,5-d']dipyrimidine-4,6(1*H*,7*H*)-dione **(6a)**

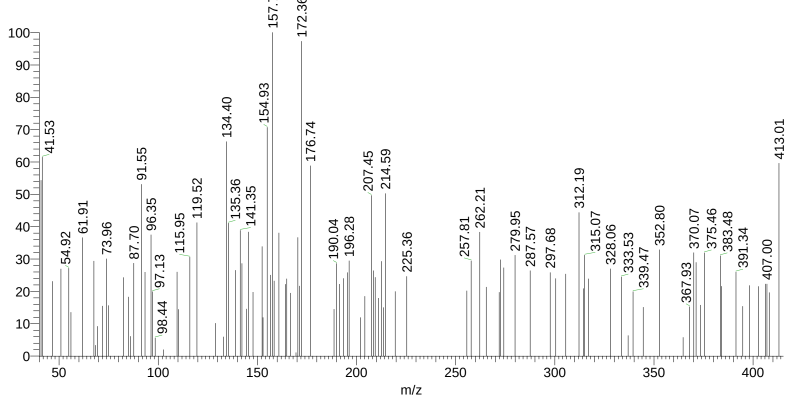

1,9-diethyl-5-(4-nitrophenyl)-2,8-dithioxo-2,3,5,8,9,10-hexahydropyrido[2,3-d:6,5-d']dipyrimidine-4,6(1*H*,7*H*)-dione **(6b)**

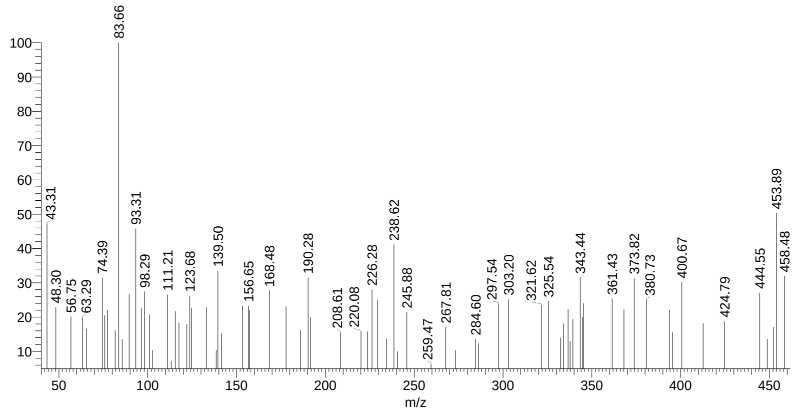

5-(4-methoxyphenyl)-1,9-dipropyl-5,10-dihydropyrido[2,3-d:6,5-d']dipyrimidine-2,4,6,8(1*H*,3*H*,7*H*,9*H*)-tetraone **(6c)**

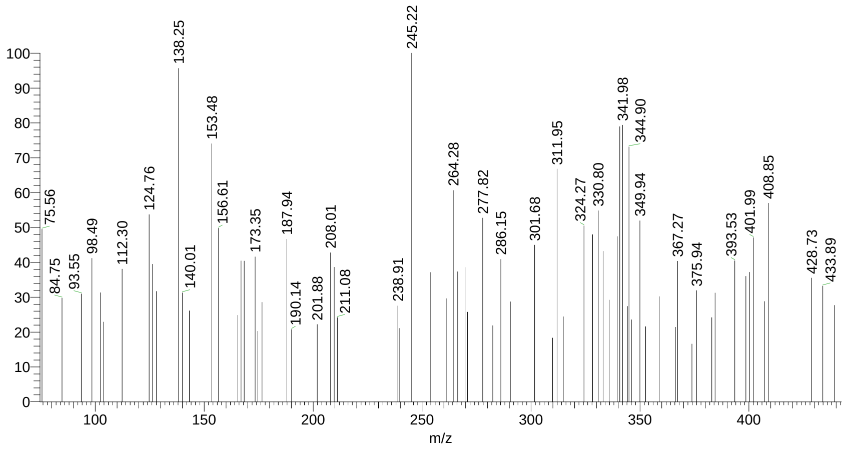

1,9-dipropyl-5,10-dihydropyrido[2,3-d:6,5-d']dipyrimidine-2,4,6,8(1*H*,3*H*,7*H*,9*H*)-tetraone **(6d)**

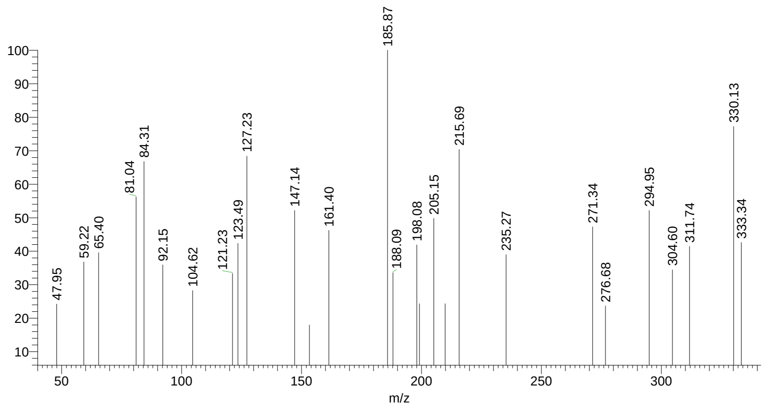

5-(4-chlorophenyl)-1,9-diethyl-2,8-dithioxo-2,3,8,9-tetrahydropyrido[2,3-d:6,5-d']dipyrimidine-4,6(1H,7*H*)-dione **(7a)**

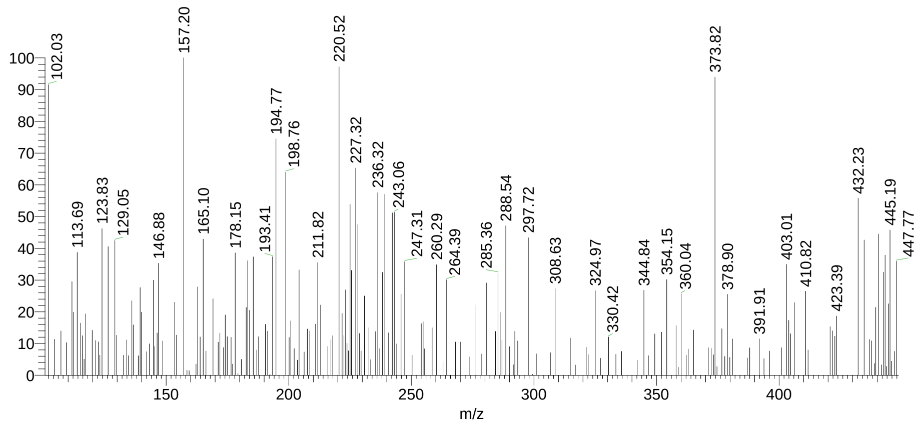

1,9-diethyl-5-(4-methoxyphenyl)-2,8-dithioxo-2,3,8,9-tetrahydropyrido[2,3-d:6,5-d']dipyrimidine-4,6(1*H*,7*H*)-dione **(7b)**

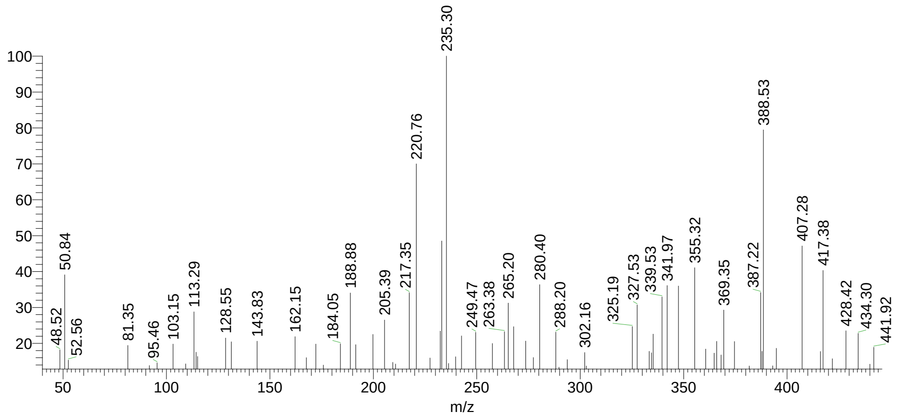

1,9-diethyl-5-(thiophen-2-yl)-2,8-dithioxo-2,3,8,9-tetrahydropyrido[2,3-d:6,5-d']dipyrimidine-4,6(1H,7H)-dione **(7c)**

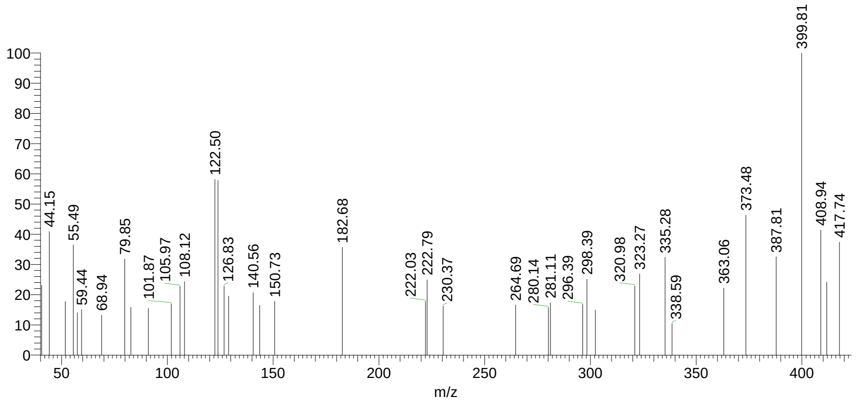

S5. HPLC analysis

HPLC Chromatograph Report

Acquired by User Me
Sample Name 5A
Injection Volume 25 ul
Data File Name C:\ Chromatograms \5A.Xml

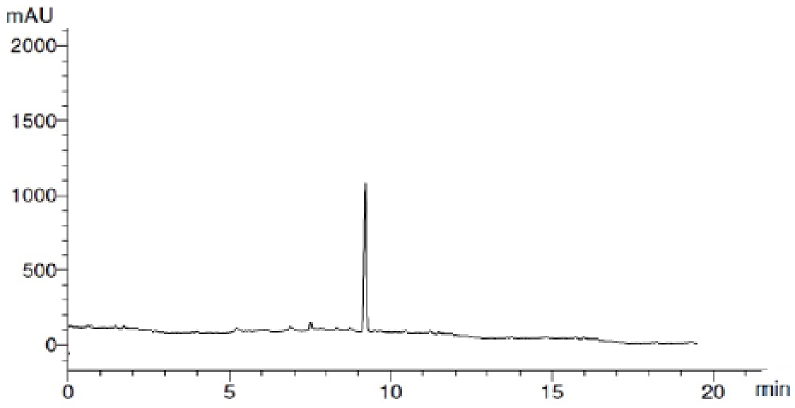

RT#	Compound	Area %
5.0	UnKnown	0.10
7.0	UnKnown	0.09
7.8	UnKnown	0.16
9.0	5a	99.65

HPLC Chromatograph Report

Acquired by	User Me
Sample Name	5M
Injection Volume	25 ul
Data File Name	C:\ Chromatograms \5M.Xml

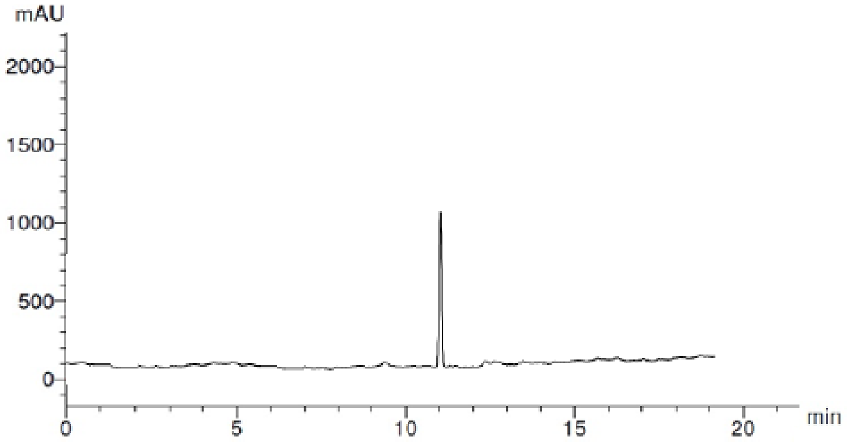

RT#	Compound	Area %
9.1	UnKnown	0.11
11.0	5M	99.71
12.0	UnKnown	0.18

HPLC Chromatograph Report

Acquired by	User Me
Sample Name	5b
Injection Volume	25 ul
Data File Name	C:\ Chromatograms \5b.Xml

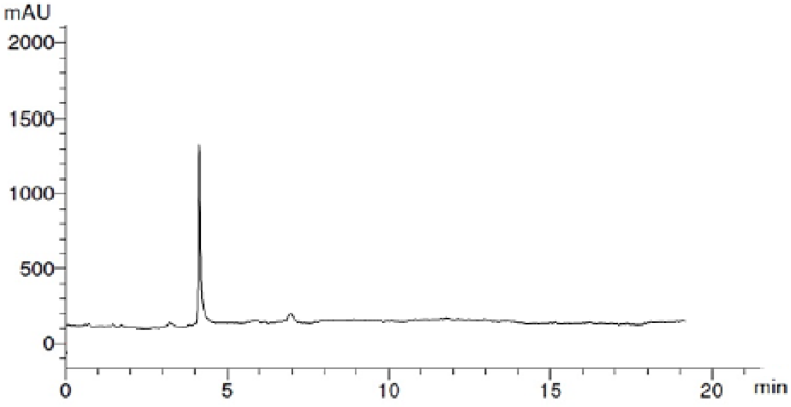

RT#	Compound	Area %
3.1	UnKnown	0.20
4.0	5b	99.65
7.0	UnKnown	0.15

HPLC Chromatograph Report

Acquired by User Me
Sample Name 5F
Injection Volume 25 ul
Data File Name C:\ Chromatograms \5F.Xml

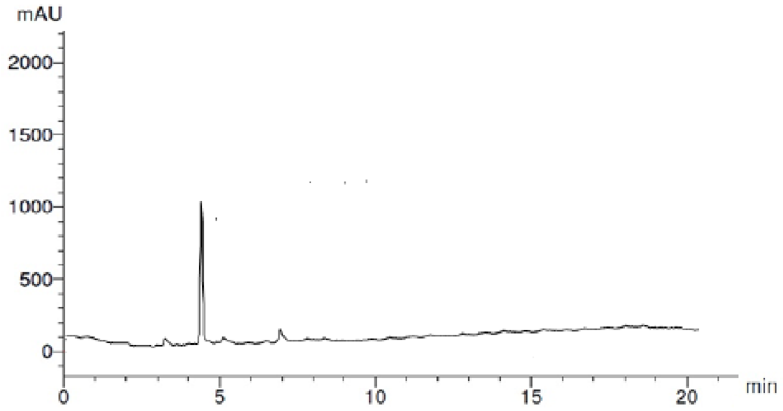

RT#	Compound	Area %
3.1	UnKnown	0.35
4.8	5F	99.00
5.1	UnKnown	0.25
7.0	UnKnown	0.40

HPLC Chromatograph Report

Acquired by User Me
Sample Name 5I
Injection Volume 25 ul
Data File Name C:\ Chromatograms \5I.Xml

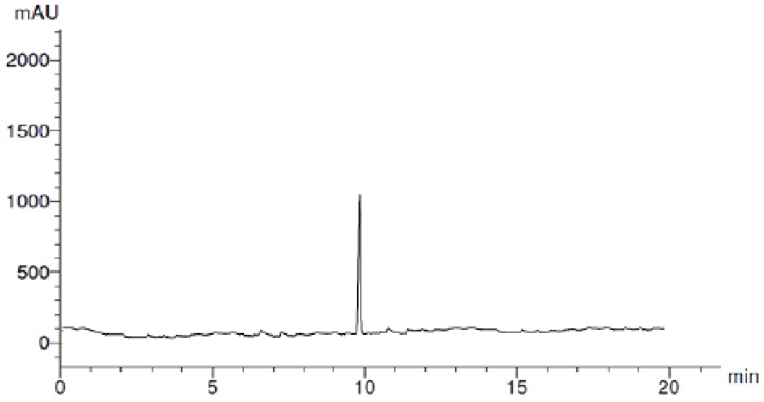

RT#	Compound	Area %
6.7	UNKnown	0.18
7.2	UNKnown	0.10
10.0	5I	99.58
11.0	UNKnown	0.14

HPLC Chromatograph Report

Acquired by User Me
Sample Name 5K
Injection Volume 25 ul
Data File Name C:\ Chromatograms \5K.Xml

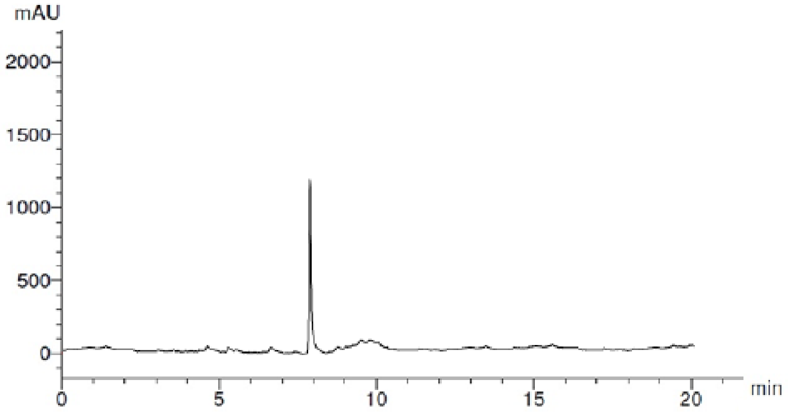

RT#	Compound	Area %
4.7	UnKnown	0.10
5.1	UnKnown	0.14
8.0	5K	99.53
10.0	UnKnown	0.23

Reference

[1] M.A. Elkady, A.S. Doghish, A. Elshafei, M.M. Elshafey, MicroRNA-567 inhibits cell proliferation and induces cell apoptosis in A549 NSCLC cells by regulating cyclin-dependent kinase 8, Saudi Journal of Biological Sciences 28(4) (2021) 2581-2590.
